# Salivary microbiota and clinical periodontal measures predicting cardiometabolic disease mortality: A nationwide survey

**DOI:** 10.1002/jper.11395

**Published:** 2025-10-10

**Authors:** Hamdi S. Adam, Weihua Guan, Abigail J. Johnson, Sanaz Sedaghat, Charlene Goh, James S. Pankow, Ryan T. Demmer

**Affiliations:** ^1^ Division of Epidemiology and Community Health University of Minnesota School of Public Health Minneapolis Minnesota USA; ^2^ Division of Biostatistics and Health Data Science University of Minnesota School of Public Health Minneapolis Minnesota USA; ^3^ Faculty of Dentistry National University of Singapore Singapore Singapore; ^4^ Division of Epidemiology Department of Quantitative Health Sciences Mayo Clinic Rochester Minnesota USA

**Keywords:** epidemiology, microbiota, mortality, periodontitis, prospective studies, Type 2 diabetes mellitus

## Abstract

**Background:**

Although periodontitis and oral microbiota are linked to cardiometabolic diseases (CMD), it is unclear if they similarly predict CMD mortality. We compared the predictive ability of salivary microbiota and periodontal disease measures for CMD mortality in the National Health and Nutrition Examination Survey (NHANES).

**Methods:**

We included 5,037 adults aged ≥30 years (mean age [± standard deviation (SD)]: 48[± 14]; 50% male) from the 2009–2010 and 2011–2012 NHANES cycles. We used 16S rRNA sequencing data from saliva to operationalize microbial composition and diversity. We calculated the relative abundance log‐ratio of *Treponema* (linked with periodontal disease) to *Corynebacterium* (linked with periodontal health) to compute the Microbial Indicator of Periodontitis (MIP). Interproximal periodontal probing depth and clinical attachment loss were measured from periodontal examinations. Mortality was ascertained through 2019. Survey‐weighted Cox models regressed mortality rates on MIP, microbial diversity, and periodontal measures to estimate hazard ratios and 95% confidence intervals (HR [95% CI]).

**Results:**

Over 8.8 median follow‐up years, there were 81 CMD and 267 all‐cause deaths. After multivariable adjustment, MIP was associated with increased CMD mortality risk (HR per 1‐SD: 2.10 [1.30–3.38]). Neither microbial diversity nor periodontitis measures were associated with CMD mortality. MIP was associated with periodontitis in multivariable modeling (risk ratio per 1‐SD: 1.29 [1.22–1.39]).

**Conclusions:**

In a nationally representative cohort, greater baseline salivary *Treponema* to *Corynebacterium* ratio predicted increased CMD mortality risk, while microbial diversity metrics and periodontal parameters were not significantly associated with CMD mortality. Longitudinal studies that further contextualize the oral microbiota are warranted.

**Plain Language Summary:**

Bacteria in the mouth that cause gum disease are linked to cardiometabolic diseases (e.g., diabetes, cardiovascular disease, kidney disease). However, it is not well understood if bacteria of the mouth can predict the risk of death due to cardiometabolic diseases. We used data from a nationwide survey of US adults to explore whether bacteria from saliva, collected from a single time point, are associated with the future risk of cardiometabolic disease death. We found that people with higher levels of gum disease bacteria were more likely to die from cardiometabolic diseases. Future studies are needed to better understand the role of gum disease bacteria in the development of cardiometabolic diseases and the risk of death.

## 1 INTRODUCTION

Type 2 diabetes is a major contributor to chronic comorbidities, reduced quality of life, and healthcare costs, as an estimated 400,000 deaths in the United States were attributed to diabetes in 2021.^1^ Given its pervasiveness, diabetes risk factor management is critical in reducing cardiometabolic disease (CMD) mortality risk.^2^ One such emerging risk factor of type 2 diabetes is periodontal disease.

Periodontal disease involves chronic inflammation of the periodontium caused by microbial shifts of the subgingival biofilm.^3^ Oral microbiota and virulence factors involved in subgingival dysbiosis are hypothesized to induce extra‐oral disruptions of glucose metabolism and systemic inflammation, potentially contributing to diabetogenesis.^4^, ^5^ Previous studies have linked periodontal microbial profiles to prediabetes and insulin resistance.^6^, ^7^ Periodontal disease is also associated with incident diabetes and cardiovascular disease,^8^, ^9^ as well as cardiovascular and all‐cause mortality events.^10^, ^11^, ^12^, ^13^ Recent studies suggest greater oral microbial diversity is associated with reduced cardiovascular disease and all‐cause mortality risks, independent of periodontal disease.^14^, ^15^, ^16^ Despite growing evidence, it remains unclear whether oral microbiome characteristics and microbiota related to periodontal disease are associated with CMD mortality, or whether they enhance the prediction of CMD mortality beyond traditional periodontal measures.

We analyzed data from the National Health and Nutrition Examination Survey (NHANES) to investigate whether baseline measures of salivary microbiota are associated with CMD mortality risk, and whether salivary microbiome indices predict CMD mortality similarly to clinical periodontal disease parameters. We hypothesize that (i) salivary microbial measures are associated with CMD mortality risk, (ii) a poorer periodontal disease profile is associated with increased CMD mortality risk, and (iii) the strength of associations between salivary microbial indices and periodontal measures in predicting CMD mortality is comparable.

## 2 MATERIALS AND METHODS

### 2.1 Study background and design

NHANES is a nationwide health and nutrition assessment of the non‐institutionalized US population. Participants were randomly selected through a multi‐stage sampling strategy.^17^, ^18^ Enrolled participants then underwent at‐home health interviews and physical and laboratory assessments in mobile examination clinics (MECs).^19^

We used data from the 2009–2010 and 2011–2012 survey cycles for our prospective cohort study, as oral microbiome data were available during those periods. From a baseline cohort of 20 293, we excluded participants (i) who were aged <18 years or pregnant (*n* = 8027), (ii) did not provide a saliva sample, had rarefied α‐diversity data below the 10 000 sequence reads per sample threshold, or did not undergo periodontal examination (*n* = 7002), and (iii) were ineligible for mortality data linkage or were missing key covariates (*n* = 227). This produced a study sample of *n* = 5037 (see Figure S1 in online *Journal of Periodontology*). This study used the Strengthening the Reporting of Observational Studies in Epidemiology (STROBE) guidelines (see Supplemental Material A in online *Journal of Periodontology*).^20^

### 2.2 Ethics statement

All participants provided written informed consent prior to enrollment. Procedures and protocols were approved by the Institutional Review Board of the Centers for Disease Control and Prevention.

### 2.3 Salivary microbiota data

Saliva samples were collected in MECs from participants ≤69 years old during the 2009–2010 and 2011–2012 cycles. Deidentified saliva were transported to the University of California, San Diego where bacterial DNA were processed via 16S rRNA sequencing (see Supplemental Material 1 in online *Journal of Periodontology*).^21^, ^22^ Three microbiota indices were produced: relative abundances of microbiota, which represent the *proportions* of microbiota present in a saliva sample; β‐diversity, which quantifies microbial similarities *between* saliva samples; and α‐diversity, which measures microbial diversity within a given saliva sample.^23^ Three dataset formats were made publicly available by NHANES: amplicon sequence variants (ASV) tables for taxonomic abundances, distance matrices for β‐diversity metrics, and tabular datasets for α‐diversity metrics.

We used principal coordinates analyses (PCoA) on non‐compositional distance matrices to calculate salivary microbial dissimilarity for the following β‐diversity metrics: unweighted UniFrac (considers microbial phylogenies), weighted UniFrac (incorporates both phylogenies and relative abundances), and Bray–Curtis dissimilarity (uses only relative abundances).^23^ We also performed PCoA on ASV tables to compute Aitchison Distance, a compositional β‐diversity metric.^24^ First axis β‐diversity PCoA values were standardized into *z*‐scores and categorized into tertiles for analyses (T1, T2, T3). We then calculated mean salivary α‐diversities for the following metrics: observed number of ASVs (measures microbial richness), Shannon diversity index, and Inverse Simpson index (both estimate evenness and richness), and Faith's phylogenetic diversity (incorporates microbial phylogenies).^23^ Mean salivary α‐diversities were also standardized into *z*‐scores and categorized into tertiles. Lastly, we calculated the microbial indicator of periodontitis (MIP) using relative abundance data. Developed previously as a biomarker of early subgingival biofilm dysbiosis, MIP was computed by taking the natural‐log ratio of *Treponema* (genus associated with diseased periodontal pockets) to *Corynebacterium* relative abundances (genus linked with periodontal health).^7^ We added 1 × 10^−6^ to the numerator and denominator of the MIP ratio to avoid division‐by‐zero errors. We standardized MIP into z‐scores and categorized it into tertiles.

### 2.4 Clinical periodontal measures

Participants aged ≥30 years underwent full‐mouth periodontal examinations in MECs (see Supplemental Material 2 in online *Journal of Periodontology*).^25^ Trained and calibrated dental examiners used periodontal probes* to measure gingival recessions and periodontal probing depths at six sites/tooth across all available teeth, excluding third molars.^25^ Examiners calculated clinical attachment loss as the difference between periodontal probing depths and gingival recession values.^25^

We computed mean interproximal periodontal probing depth (I‐PPD) and interproximal clinical attachment loss (I‐CAL) from mesial and distal sites across all available teeth for each participant. Interproximal parameters were selected as robust indicators of periodontal disease. Mean I‐PPD and I‐CAL were operationalized as z‐scores and tertiles. Periodontal disease was defined via the Centers for Disease Control/American Academy of Periodontology classification (CDC/AAP; grouped as *healthy/mild*, *moderate*, *severe*).^25^ For correlations between continuous microbial indices and periodontal parameters, see Table S1 in online *Journal of Periodontology*.

### 2.5 Mortality outcomes

Deaths from baseline (2009–2010, 2011–2012) through December 31, 2019, were ascertained in NHANES with National Death Index linkages, and were classified using the International Statistical Classification of Diseases, Injuries, and Causes of Death, 10th Edition.^26^ Our primary outcome is CMD mortality, defined as deaths due to diabetes, diseases of the heart, cerebrovascular diseases, or kidney diseases as the leading cause.^27^ Our secondary outcome is all‐cause mortality. For descriptive analyses, deaths due to cancer, respiratory illnesses, influenza, pneumonia, or Alzheimer's disease were classified as chronic inflammatory disease deaths (INFL), while non‐disease deaths were labeled as deaths from other causes.

### 2.6 Baseline covariates

Sociodemographics and smoking history were ascertained from at‐home interviews.^19^, ^28^ Body mass index (BMI) was collected from physical exams.^29^ Using the *dietaryindex* package in R, the Alternative Healthy Eating Index (AHEI) was calculated from 24‐h food recalls.^30^ We estimated weekly physical activity levels via metabolic equivalent of tasks scores (MET) categorized per Physical Activity Guidelines for Americans.^31^, ^32^ Mean systolic and diastolic blood pressures were measured at MECs. We defined prevalent hypertension as systolic blood pressure ≥130 mm Hg, diastolic blood pressure ≥80 mm Hg, or self‐reported anti‐hypertensive medication use.^33^ Total cholesterol and glycated hemoglobin A1c (HbA1c) were measured from non‐fasting blood samples.^34^, ^35^ We defined prediabetes as HbA1c of 5.7%–6.5% or self‐reported prediabetes diagnosis, and diabetes as HbA1c ≥6.5% or self‐reported diabetes diagnosis.^36^ We determined high cholesterol as total cholesterol ≥200 mg/dL.^37^ Prevalent cardiovascular disease or kidney disease was defined via self‐report. For additional covariate information, see Supplemental Material 3 in online *Journal of Periodontology*.

### 2.7 Statistical analysis

We first assessed unweighted baseline participant characteristics across mortality categories. Next, we conducted median imputations for missing continuous covariates. Upon observing no violation of the proportional hazards assumption, we proceeded with survey‐weighted multivariable Cox regression to estimate hazard ratios and 95% confidence intervals (HR [95% CI]) of CMD and all‐cause mortality across MIP, periodontal parameters, first axis PCoA β‐diversity, and α‐diversity in our main analyses. Natural‐log HRs (log‐HR [95% CIs]) were also computed. We adjusted for survey cycle (Model 1), sociodemographics (Model 2 = Model 1 + age, sex, race/ethnicity, education, income), and health behaviors (Model 3 = Model 2 + BMI, AHEI, physical activity, smoking). In Model 4, we additionally adjusted for CDC/AAP (when exposure = MIP, first axis PCoA β‐diversity, or α‐diversity) and Shannon index (when exposure = periodontal measure). Also, we further adjusted for cardiovascular risk factors in Model 5 (Model 4 + HbA1c, systolic blood pressure, total cholesterol). In secondary analyses, we examined whether MIP was associated with the prevalence of moderate/severe periodontal disease via survey‐weighted robust‐variance Poisson regression models, and if associations from the main analyses were modified by periodontal status (via CDC/AAP). From there, we profiled the salivary microbiota by (i) performing PCoA to explore β‐diversity dissimilarity across periodontal disease (CDC/AAP) and mortality status (CMD, INFL, other, alive);^38^ (ii) examining α‐diversity distributions by periodontal disease and mortality status using pairwise comparisons and global statistical tests; and (iii) computing normalized relative abundances of microbial phyla across levels of Shannon index, periodontal disease categories, and by mortality subtypes. Finally, we assessed microbial relative abundance patterns using the analysis of compositions of microbiomes with bias correction (ANCOMBC) R package.^39^ Through this approach, we identified microbial taxa that were differentially abundant by periodontal disease (moderate/severe vs. healthy/mild), determined by false discovery rates (FDR) *q*‐values < 0.05 in multivariable modeling.

Analyses were completed in PC‐SAS v9.4 and R v4.3.2. *p*‐values < 0.05 indicated statistical significance. For analysis descriptions, see Supplemental Material 4 in online *Journal of Periodontology*.

## 3 RESULTS

### 3.1 Participant characteristics

Among 5 037 participants, mean (standard deviation [± SD]) age at baseline was 48.0 [± 14.2] years, and 50.2% were male. Race/ethnicity distributions were 16.7% Mexican American, 10.2% Other Hispanic, 38.8% Non‐Hispanic White, 23.2% Non‐Hispanic Black, and 11.1% Other self‐reported race/ethnicities.

Baseline participant characteristics are shown in Table 1. Across 8.8 years of median follow‐up, 267 deaths occurred, of which 81 were CMD mortality and 104 were INFL mortality. Compared with those alive throughout follow‐up, participants who died from CMD or INFL were on average 9 years older, more likely to be male, have less education and income, history of smoking, and poorer periodontal measures at baseline. Individuals who died from CMD were more likely to have obesity, poorer diet quality, hypertension, diabetes, and moderate to severe periodontal disease at baseline than participants who died from INFL. Compared with participants who remained alive, those who died from CMD or INFL had moderately lower mean observed ASVs and Shannon index at baseline, while Inverse Simpson and Faith's Phylogenetic diversity indices were similar by mortality status.

**TABLE 1:** Unweighted participant characteristics at baseline by mortality categories (*n* = 5037; NHANES 2009–2010, 2011–2012).

		Mortality status		Leading causes of death		
	Total *N* = 5037	Alive *n* = 4770	Died *n* = 267	Cardiometabolic disease (CMD) *n* = 81	Chronic inflammatory disease (INFL) *n* = 104	Other causes *n* = 82
Survey cycle						
2009–2010	2568 (51.0%)	2427 (50.9%)	141 (52.8%)	38 (46.9%)	66 (63.5%)	37 (45.1%)
2011–2012	2469 (49.0%)	2343 (49.1%)	126 (47.2%)	43 (53.1%)	38 (36.5%)	45 (54.9%)
Follow‐up time (years)						
Median [min, max]	8.83 [0.08, 11.08]	9.00 [6.75, 11.08]	5.42 [0.08, 10.75]	5.83 [0.08, 10.75]	5.42 [0.08, 10.75]	5.46 [0.17, 10.17]
Age (years)						
Mean (SE)	48.0 (0.2)	47.6 (0.2)	55.2 (0.6)	56.5 (1.1)	56.6 (0.8)	51.9 (1.1)
Median [min, max]	48.0 [30.0, 69.0]	47.0 [30.0, 69.0]	57.0 [31.0, 69.0]	60.0 [31.0, 69.0]	58.0 [33.0, 69.0]	52.0 [31.0, 69.0]
Sex						
Male	2527 (50.2%)	2356 (49.4%)	171 (64.0%)	53 (65.4%)	64 (61.5%)	54 (65.9%)
Female	2510 (49.8%)	2414 (50.6%)	96 (36.0%)	28 (34.6%)	40 (38.5%)	28 (34.1%)
Race/ethnicity						
Mexican American	839 (16.7%)	801 (16.8%)	38 (14.2%)	10 (12.3%)	16 (15.4%)	12 (14.6%)
Other Hispanic	514 (10.2%)	496 (10.4%)	18 (6.7%)	6 (7.4%)	8 (7.7%)	4 (4.9%)
Non‐Hispanic White	1955 (38.8%)	1839 (38.6%)	116 (43.4%)	26 (32.1%)	54 (51.9%)	36 (43.9%)
Non‐Hispanic Black	1168 (23.2%)	1086 (22.8%)	82 (30.7%)	34 (42.0%)	21 (20.2%)	27 (32.9%)
Other self‐reported race/ethnicity	561 (11.1%)	548 (11.5%)	13 (4.9%)	5 (6.2%)	5 (4.8%)	3 (3.7%)
Education						
Some high school	1226 (24.3%)	1132 (23.7%)	94 (35.2%)	30 (37.0%)	30 (28.8%)	34 (41.5%)
High school/GED	1065 (21.1%)	1007 (21.1%)	58 (21.7%)	21 (25.9%)	25 (24.0%)	12 (14.6%)
Some college	1403 (27.9%)	1333 (27.9%)	70 (26.2%)	20 (24.7%)	25 (24.0%)	25 (30.5%)
College graduate or more	1343 (26.7%)	1298 (27.2%)	45 (16.9%)	10 (12.3%)	24 (23.1%)	11 (13.4%)
Annual family income						
<$20 000	1108 (22.0%)	1002 (21.0%)	106 (39.7%)	36 (44.4%)	37 (35.6%)	33 (40.2%)
$20 000–75 000	2544 (50.5%)	2421 (50.8%)	123 (46.1%)	38 (46.9%)	50 (48.1%)	35 (42.7%)
>$75 000	1385 (27.5%)	1347 (28.2%)	38 (14.2%)	7 (8.6%)	17 (16.3%)	14 (17.1%)
Body mass index (BMI)						
<25 kg/m^2^	1269 (25.2%)	1202 (25.2%)	67 (25.1%)	16 (19.8%)	23 (22.1%)	28 (34.1%)
25–30 kg/m^2^	1731 (34.4%)	1648 (34.5%)	83 (31.1%)	25 (30.9%)	37 (35.6%)	21 (25.6%)
>30 kg/m^2^	2010 (39.9%)	1896 (39.7%)	114 (42.7%)	40 (49.4%)	41 (39.4%)	33 (40.2%)
Missing	27 (0.5%)	24 (0.5%)	3 (1.1%)	0 (0%)	3 (2.9%)	0 (0%)
Alternative health eating index (AHEI)						
Mean (SE)	38.0 (0.2)	38.2 (0.2)	34.9 (0.7)	32.8 (1.1)	36.2 (1.3)	35.6 (1.3)
Median [min, max]	37.3 [2.61, 86.2]	37.6 [2.61, 86.2]	34.2 [10.2, 69.9]	32.2 [12.6, 55.9]	34.1 [10.2, 69.9]	35.8 [13.6, 59.1]
Missing	231 (4.6%)	219 (4.6%)	12 (4.5%)	1 (1.2%)	7 (6.7%)	4 (4.9%)
Physical activity level						
<500 MET min/week	3276 (65.0%)	3120 (65.4%)	156 (58.4%)	51 (63.0%)	54 (51.9%)	51 (62.2%)
500–1000 MET min/week	446 (8.9%)	427 (9.0%)	19 (7.1%)	4 (4.9%)	8 (7.7%)	7 (8.5%)
>1000 MET min/week	140 (2.8%)	136 (2.9%)	4 (1.5%)	2 (2.5%)	1 (1.0%)	1 (1.2%)
Missing	1175 (23.3%)	1087 (22.8%)	88 (33.0%)	24 (29.6%)	41 (39.4%)	23 (28.0%)
Smoking history						
Never smoker	2812 (55.8%)	2703 (56.7%)	109 (40.8%)	39 (48.1%)	38 (36.5%)	32 (39.0%)
Former smoker	1136 (22.6%)	1073 (22.5%)	63 (23.6%)	17 (21.0%)	30 (28.8%)	16 (19.5%)
Current smoker	1089 (21.6%)	994 (20.8%)	95 (35.6%)	25 (30.9%)	36 (34.6%)	34 (41.5%)
Prevalent periodontal disease (CDC/AAP classification)						
Healthy	2430 (48.2%)	2362 (49.5%)	68 (25.5%)	18 (22.2%)	27 (26.0%)	23 (28.0%)
Mild	351 (7.0%)	331 (6.9%)	20 (7.5%)	9 (11.1%)	6 (5.8%)	5 (6.1%)
Moderate	1626 (32.3%)	1510 (31.7%)	116 (43.4%)	37 (45.7%)	44 (42.3%)	35 (42.7%)
Severe	630 (12.5%)	567 (11.9%)	63 (23.6%)	17 (21.0%)	27 (26.0%)	19 (23.2%)
Interproximal CAL (mm)						
Mean (SE)	1.80 (0.02)	1.76 (0.02)	2.51 (0.10)	2.55 (0.18)	2.49 (0.16)	2.50 (0.18)
Median [min, max]	1.44 [0.01, 12.33]	1.41 [0.01, 12.33]	2.09 [0.47, 11.50]	2.15 [0.47, 9.24]	2.02 [0.51, 7.69]	2.06 [0.61, 11.50]
Interproximal PPD (mm)						
Mean (SE)	1.85 (0.01)	1.83 (0.01)	2.09 (0.05)	2.13 (0.10)	2.04 (0.08)	2.12 (0.09)
Median [min, max]	1.69 [0.11, 6.08]	1.67 [0.11, 6.08]	1.95 [0.46, 5.41]	1.97 [0.46, 5.39]	1.90 [0.57, 5.41]	2.06 [0.48, 3.88]
Prevalent hypertension						
Yes	2420 (48.0%)	2240 (47.0%)	180 (67.4%)	60 (74.1%)	67 (64.4%)	53 (64.6%)
No	2447 (48.6%)	2366 (49.6%)	81 (30.3%)	20 (24.7%)	34 (32.7%)	27 (32.9%)
Missing	170 (3.4%)	164 (3.4%)	6 (2.2%)	1 (1.2%)	3 (2.9%)	2 (2.4%)
Mean systolic blood pressure (mm Hg)						
Mean (SE)	122.2 (0.2)	121.8 (0.2)	130 (1.3)	132.4 (2.6)	128.5 (2.0)	129.2 (2.4)
Median [min, max]	120.0 [80.0, 225.3]	119.3 [80.0, 225.3]	127 [83.3, 219.3]	127.7 [94.0, 207.3]	125.3 [83.3, 190.0]	123.3 [96.0, 219.3]
Missing	234 (4.6%)	217 (4.5%)	17 (6.4%)	5 (6.2%)	8 (7.7%)	4 (4.9%)
Mean diastolic blood pressure (mm Hg)						
Mean (SE)	72.9 (0.2)	72.8 (0.2)	74.6 (0.9)	75.0 (1.6)	73.1 (1.3)	76.3 (1.6)
Median [min, max]	73.3 [0.0, 120.0]	72.7 [0.0, 120.0]	74.0 [19.3, 118.7]	74.0 [45.3, 116.0]	73.0 [39.3, 118.7]	75.0 [19.3, 116.7]
Missing	234 (4.6%)	217 (4.5%)	17 (6.4%)	5 (6.2%)	8 (7.7%)	4 (4.9%)
Prevalent high cholesterol						
Yes	2315 (46.0%)	2215 (46.4%)	100 (37.5%)	33 (40.7%)	38 (36.5%)	29 (35.4%)
No	2485 (49.3%)	2344 (49.1%)	141 (52.8%)	45 (55.6%)	50 (48.1%)	46 (56.1%)
Missing	237 (4.7%)	211 (4.4%)	26 (9.7%)	3 (3.7%)	16 (15.4%)	7 (8.5%)
Total cholesterol (mg/dL)						
Mean (SE)	200.4 (0.6)	200.8 (0.6)	194 (3.0)	194.5 (5.2)	195.7 (5.1)	193.0 (5.2)
Median [min, max]	198.0 [75.0, 523.0]	198.0 [92.0, 523.0]	190 [75.0, 389.0]	190.5 [75.0, 389.0]	192.5 [99.0, 327.0]	188.0 [93.0, 327.0]
Missing	237 (4.7%)	211 (4.4%)	26 (9.7%)	3 (3.7%)	16 (15.4%)	7 (8.5%)
Diabetes status						
Diabetes	732 (14.5%)	658 (13.8%)	74 (27.7%)	38 (46.9%)	19 (18.3%)	17 (20.7%)
Prediabetes	1377 (27.3%)	1290 (27.0%)	87 (32.6%)	23 (28.4%)	37 (35.6%)	27 (32.9%)
Healthy	2759 (54.8%)	2670 (56.0%)	89 (33.3%)	18 (22.2%)	36 (34.6%)	35 (42.7%)
Missing	169 (3.4%)	152 (3.2%)	17 (6.4%)	2 (2.5%)	12 (11.5%)	3 (3.7%)
HbA1c (%)						
Mean (SE)	5.81 (0.02)	5.78 (0.02)	6.30 (0.11)	6.81 (0.20)	6.02 (0.15)	6.13 (0.21)
Median [min, max]	5.50 [3.60, 17.80]	5.50 [3.60, 17.80]	5.80 [4.30, 16.90]	6.10 [4.30, 12.70]	5.70 [4.30, 12.60]	5.70 [4.60, 16.90]
Missing	186 (3.7%)	166 (3.5%)	20 (7.5%)	3 (3.7%)	14 (13.5%)	3 (3.7%)
Prevalent cardiovascular or cardiometabolic comorbidity^a^ (%)						
Yes	364 (7.2%)	316 (6.6%)	48 (18.0%)	20 (24.7%)	18 (17.3%)	10 (12.2%)
No	4637 (92.1%)	4423 (92.7%)	214 (80.1%)	57 (70.4%)	86 (82.7%)	71 (86.6%)
Missing	36 (0.7%)	31 (0.7%)	5 (1.9%)	4 (4.9%)	0 (0%)	1 (1.2%)
Observed ASVs						
Mean (SE)	132.6 (0.6)	132.9 (0.6)	127 (2.7)	128.5 (4.9)	122.1 (4.3)	133.3 (4.8)
Median [min, max]	127.9 [13.0, 348.2]	128.0 [15.0, 348.2]	121 [13.0, 262.0]	120.9 [35.0, 246.9]	120.6 [13.0, 251.90]	129.6 [41.0, 262.0]
Shannon diversity index						
Mean (SE)	4.65 (0.01)	4.65 (0.01)	4.54 (0.04)	4.53 (0.08)	4.52 (0.07)	4.58 (0.72)
Median [min, max]	4.69 [0.50, 6.60]	4.69 [0.50, 6.60]	4.61 [2.05, 6.39]	4.54 [2.12, 5.98]	4.57 [2.43, 6.39]	4.67 [2.05, 6.03]
Inverse Simpson index						
Mean (SE)	0.90 (0.00)	0.90 (0.00)	0.899 (0.00)	0.89 (0.01)	0.90 (0.01)	0.90 (0.01)
Median [min, max]	0.92 [0.10, 0.98]	0.92 [0.10, 0.98]	0.91 [0.48, 0.98]	0.91 [0.62, 0.97]	0.91 [0.65, 0.98]	0.91 [0.48, 0.97]
Faith's phylogenetic diversity						
Mean (SE)	14.7 (0.1)	14.8 (0.1)	14.4 (0.2)	14.5 (0.4)	14.0 (0.3)	14.8 (0.37)
Median [min, max]	14.5 [2.8, 31.3]	14.5 [2.8, 31.3]	14.2 [3.7, 27.0]	14.4 [5.9, 27.0]	14.0 [3.7, 22.9]	14.7 [6.3, 22.7]

*Note*: All statistics are derived from unweighted descriptive analyses.

Abbreviations: AHEI, alternative health eating index; ASV, amplicon sequencing variants; CAL, clinical attachment loss; CDC/AAP, Centers for Disease Control/American Academy of Periodontology; MET, metabolic equivalent of task; PPD, periodontal probing depth; SE, standard error.

^a^ Prevalent Cardiovascular or Cardiometabolic Comorbidities refer to self‐reported physician diagnosis of cardiovascular disease, cerebrovascular disease, diabetes, and/or kidney disease at baseline.

### 3.2 MIP, periodontal disease, and mortality

**FIGURE 1:**
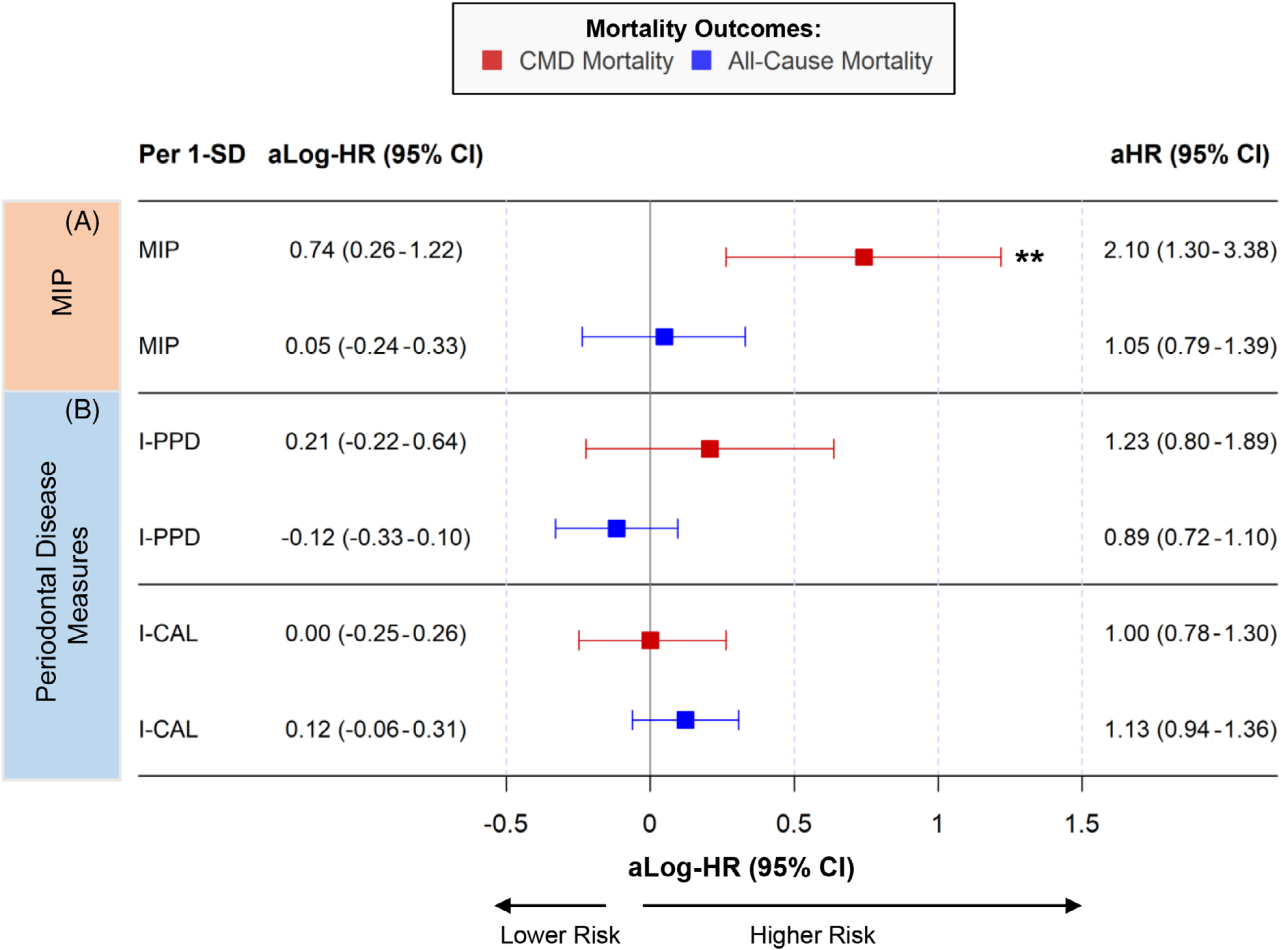
Microbial indicator of periodontitis and periodontal disease measures predicting mortality outcomes (*n* = 5037; NHANES 2009–2010, 2011–2012). Panel (A) shows point estimates of MIP predicting mortality outcomes. Panel (B) shows point estimates of periodontal disease measures (interproximal periodontal probing depth [I‐PPD] and interproximal clinical attachment loss [I‐CAL]) predicting mortality outcomes. **Indicates statistically significant log‐HR (*p*‐value < 0.05) and statistically significant Bonferroni‐adjusted *p*‐value for multiple comparisons, assuming a Type 1 error rate of 0.05 (0.05/12 comparisons = 0.00416667 adjusted significance threshold). Log‐hazard ratios and 95% confidence intervals were computed using survey‐weighted multivariable proportional hazards regression. Adjusted covariates include survey cycle, age, sex, race/ethnicity, education, income, BMI, alternative healthy eating index, physical activity, smoking, CDC/AAP^a^, Shannon index^b^, HbA1c, systolic blood pressure, and total cholesterol. ^a^CDC/AAP was adjusted in models when MIP was the independent variable. ^b^Shannon index was adjusted in models when I‐CAL and I‐PPD were the independent variables. aHR, multivariable‐adjusted hazard ratio; aLog‐HR, multivariable‐adjusted natural‐log hazard ratio; CI, 95% confidence interval; CMD, cardiometabolic disease mortality; I‐CAL, interproximal clinical attachment loss; I‐PPD, interproximal periodontal probing depth; MIP, microbial indicator of periodontitis; SD, standard deviation.

Figure 1 summarizes the multivariable‐adjusted associations between standardized MIP and periodontal disease measures in predicting mortality outcomes. Standardized MIP was positively associated with CMD mortality (HR: 2.10 [1.30–3.38]) but showed no significant patterns in predicting all‐cause mortality (HR: 1.05 [0.79–1.39]) after multivariable adjustments (Figure 1A). Categorical MIP was linearly associated with elevated CMD mortality risks (T2 vs. T1 HR: 1.38 [0.65–2.94]; T3 vs. T1 HR: 4.26 [1.32–13.71]; linear trend *p*‐value = 0.02; see Table S2 in online *Journal of Periodontology*). However, linear trends were not observed for MIP predicting all‐cause mortality (T2 vs. T1 HR: 0.83 [0.54–1.27]; T3 vs. T1 HR: 1.09 [0.58–2.03]; linear trend *p*‐value = 0.79).

In Figure 1B, standardized mean I‐PPD and I‐CAL were not associated with mortality outcomes. When assessed across tertiles, greater I‐PPD and I‐CAL showed elevated risks of mortality, including all‐cause mortality in multivariable models (I‐PPD T3 vs. T1: 1.37 [0.84–2.22]; I‐CAL T3 vs. T1: 2.10 [1.25–3.52]). However, associations were largely not statistically significant (see Table S3 in online *Journal of Periodontology*). Periodontal disease, via CDC/AAP definition, was not associated with mortality outcomes.

MIP was cross‐sectionally associated with increased prevalence of moderate/severe periodontal disease in multivariable models (prevalence ratio [95% CI] per 1‐SD MIP: 1.29 [1.22–1.39]; see Table S4 in online *Journal of Periodontology*). Subgroup analyses indicate positive associations between MIP and CMD mortality across all periodontal disease categories, with especially greater risk among participants without periodontitis or with mild periodontitis (HR per 1‐SD MIP: 2.93 [1.60–5.39]; see Table S5 in online *Journal of Periodontology*). Overall, periodontitis did not modify the association of MIP and CMD mortality (interaction *p*‐value = 0.15).

### 3.3 Salivary microbial diversity and mortality

**FIGURE 2:**
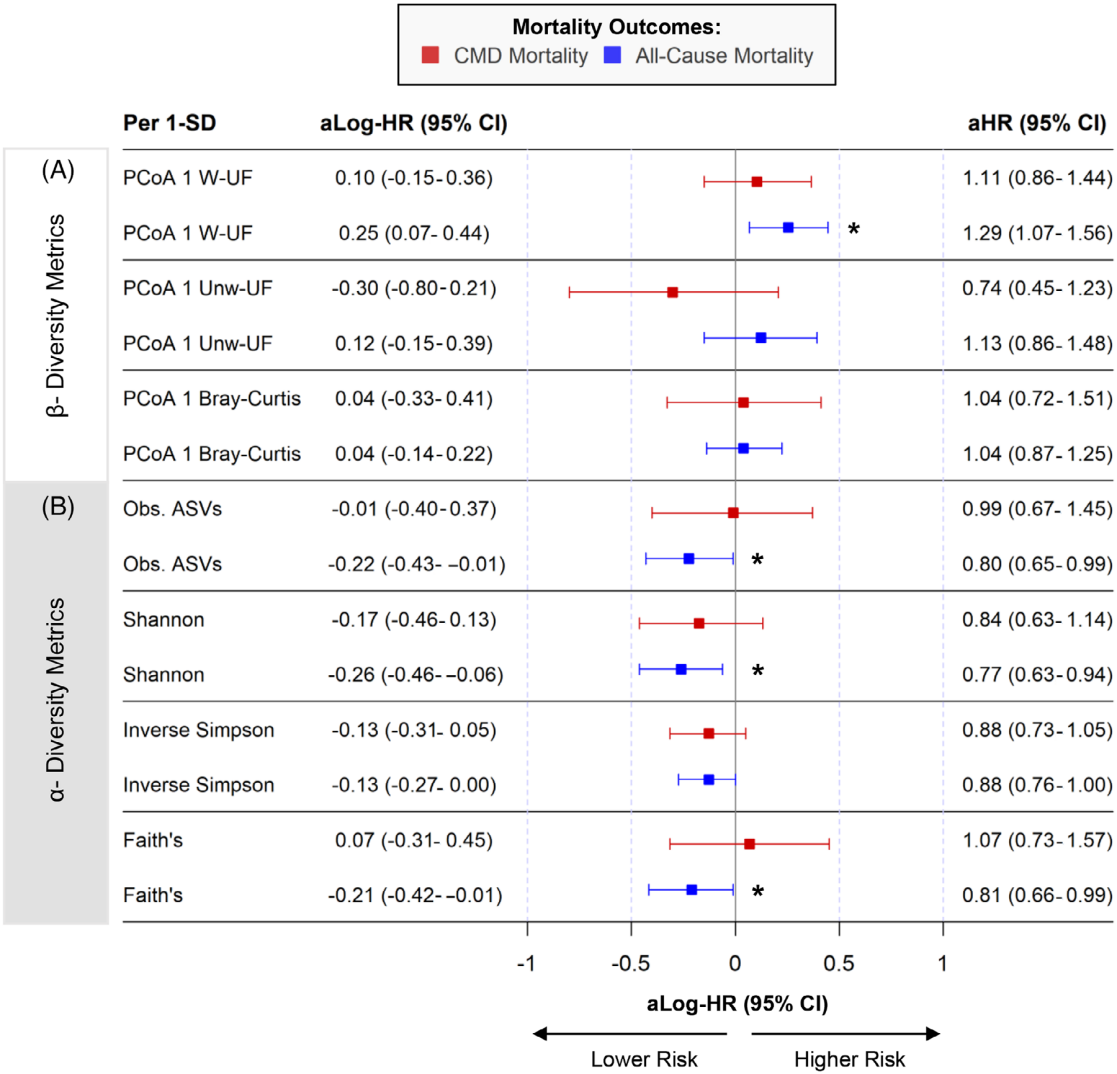
Association between salivary microbial diversity and mortality outcomes (*n* = 5037; NHANES 2009–2010, 2011–2012). Panel (A) shows point estimates of beta diversity metrics on the first PCoA Axis (weighted unifrac [W‐UF], unweighted unifrac [Unw‐UF], and Bray‐Curtis) predicting mortality outcomes. Panel (B) shows point estimates of alpha diversity metrics (observed ASVs, shannon diversity index, inverse simpson index, and faith's phylogenetic diversity) predicting mortality outcomes. *Indicates statistically significant log‐HR (*p*‐value < 0.05). Log‐hazard ratios and 95% confidence intervals were computed using survey‐weighted multivariable proportional hazards regression. Adjusted covariates include survey cycle, age, sex, race/ethnicity, education, income, BMI, alternative healthy eating index, physical activity, smoking, CDC/AAP, HbA1c, systolic blood pressure, and total cholesterol. aHR, multivariable‐adjusted hazard ratio; aLog‐HR, multivariable‐adjusted natural‐log hazard ratio; CI, 95% confidence interval; CMD, cardiometabolic disease mortality; Obs. ASVs, observed amplicon sequence variants; PCoA1, principal coordinate analysis axis 1; SD, standard deviation; Unw‐UF, unweighted UniFrac; W‐UF, weighted UniFrac.

Figure 2 visualizes the multivariable‐adjusted relationship between standardized salivary microbial diversity and mortality outcomes. β‐diversity metrics on PCoA axis 1 were not associated with CMD mortality (Figure 2A). Weighted UniFrac on the first PCoA axis per 1‐SD increment was associated with an elevated risk of all‐cause mortality (HR: 1.29 [1.07–1.56]). Tertiles of β‐diversity metrics on PCoA axis 1 were not associated with mortality outcomes after multivariable adjustments (see Table S6 in online *Journal of Periodontology*).

Standardized α‐diversity metrics were not associated with CMD mortality but showed modest inverse trends with all‐cause mortality (Figure 2B). Specifically, all‐cause mortality risk was significantly lower per 1‐SD increment of observed ASVs (HR: 0.80 [0.65–0.99]), Shannon index (HR: 0.77 [0.63–0.94]), and Faith's phylogenetic diversity (HR: 0.81 [0.66–0.99]). Multivariable models showed no meaningful associations between tertiles of α‐diversity metrics and CMD mortality (see Table S7 in online *Journal of Periodontology*). Lastly, despite covariate adjustments showing no significant association, higher tertiles of α‐diversity consistently trended toward lower all‐cause mortality risk (e.g., Shannon index T2 vs. T1 HR: 0.68 [0.40–1.18]; T3 vs. T1 HR: 0.60 [0.35–1.04]; linear trend *p*‐value = 0.07).

Higher Shannon diversity index was associated with non‐significantly lower risks of CMD mortality among those with healthy/mild and moderate periodontitis, while individuals with severe periodontitis had non‐significant increased risks (see Table S5 in online *Journal of Periodontology*). There was no evidence that periodontal disease modified the association of Shannon index and CMD mortality (interaction *p*‐value = 0.14).

### 3.4 Microbial diversity profile

**FIGURE 3:**
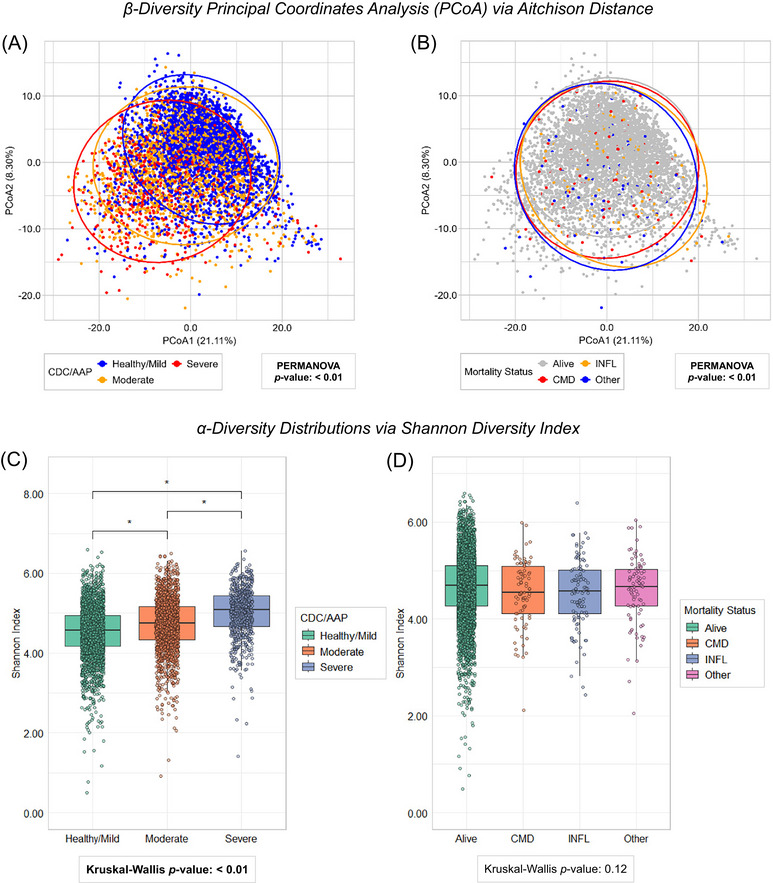
Salivary microbial diversity analysis (*n* = 5037; NHANES 2009–2010, 2011–2012). (A) PCoA of Aitchison distance by periodontal disease status. (B) PCoA of Aitchison distance by mortality status. (C) Distribution of Shannon diversity index by periodontal disease status. (D) Distribution of Shannon diversity index by mortality status. Note that Aitchison distance is a compositional β‐diversity metric. Non‐parametric Kruskal–Wallis and pairwise Mann–Whitney *U* tests were used. Statistical significance (*p*‐value < 0.05) is represented with asterisks (for pairwise comparisons) and bold font (global test). CDC/AAP, Centers for Disease Control and Prevention/American Academy of Periodontology classification system; CMD, cardiometabolic disease mortality; INFL, chronic inflammatory disease mortality; PCoA1, principal coordinate analysis first axis; PCoA2, principal coordinate analysis second axis; PERMANOVA, permutational multivariate analysis of variance.

Figure 3 depicts salivary microbial diversity across periodontal disease categories and mortality status, with each dot representing an individual participant's salivary microbiota. In β‐diversity PCoA of Aitchison's distance, 29.41% of the total variability in microbial composition between saliva samples was attributed to the first (21.11%) and the second (8.30%) PCoA axes. Participants with moderate and severe periodontal disease had more similar salivary microbial compositions compared with those of healthy or mild periodontal disease categories (Figure 3A, *p *< 0.01). Salivary microbial composition significantly differed by mortality subtype, although distinct clustering patterns were hard to visualize (Figure 3B, *p *< 0.01). Despite the lack of statistical significance, moderate clustering patterns across periodontal status and mortality status were observed with unweighted UniFrac, weighted UniFrac, and Bray‐Curtis β‐diversity metrics (see Figure S2 in online *Journal of Periodontology*).

In α‐diversity analyses, pairwise comparisons of Shannon index showed participants with moderate and severe periodontal disease had significantly more diverse salivary microbiota compared with saliva from individuals of healthy or mild periodontal disease categories (Figure 3C, global test *p *< 0.01). Mean Shannon index did not significantly differ by mortality subtype (Figure 3D, global test *p *= 0.12). Consistent patterns for periodontal disease and mortality status were exhibited across additional α‐diversity metrics (see Figure S3 in online *Journal of Periodontology*).

### 3.5 Microbial composition profile

**FIGURE 4:**
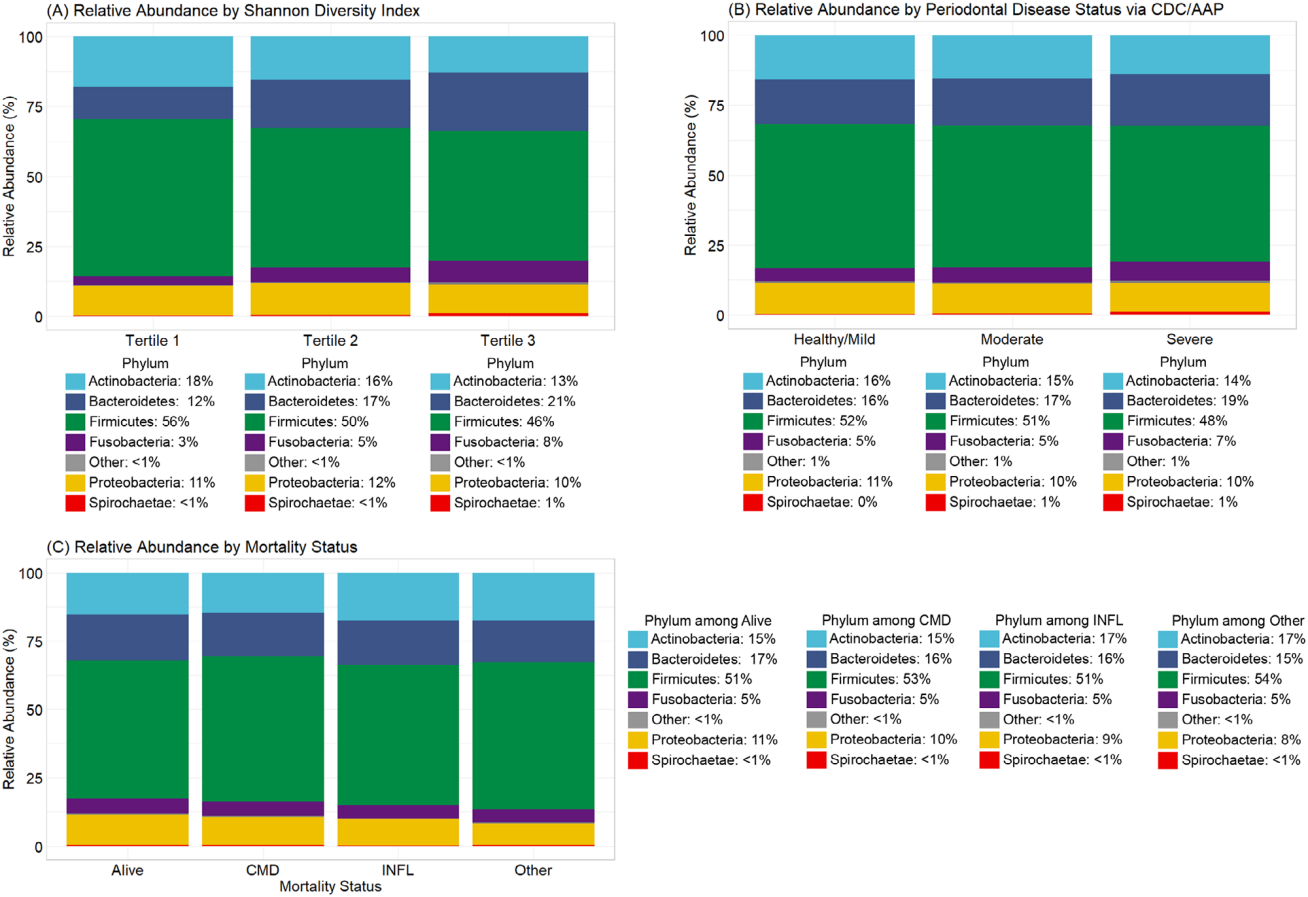
Normalized relative abundance of salivary microbiota (*n* = 5037; NHANES 2009–2010, 2011–2012). Panel (A) shows the relative abundances of microbial phyla by tertiles of shannon index alpha diversity. Panel (B) shows the relative abundances of microbial phyla by periodontal disease status via CDC/AAP. Panel (C) shows the relative abundance of microbial taxa by mortality status (alive, cardiometabolic disease mortality [CMD], chronic inflammatory disease mortality [INFL], and all other causes of death). CDC/AAP, Centers for Disease Control and Prevention/American Academy of Periodontology; CMD, cardiometabolic disease mortality; INFL, chronic inflammatory disease mortality; Other, other causes of mortality.

Phylum‐level microbial composition across key variables is presented in Figure 4. Compared with the lowest tertile, participants with higher salivary α‐diversity (via Shannon index) showed increased relative abundances of Bacteroidetes and fusobacteria, and decreased abundances of Actinobacteria and Firmicutes (Figure 4A). Taxa were relatively stable across periodontal disease categories, with subtle shifts in Actinobacteria, Bacteroidetes, and Firmicutes (Figure 4B). Lastly, minor differences in Actinobacteria, Firmicutes, and proteobacteria abundances were observed across mortality status (Figure 4C).

**FIGURE 5:**
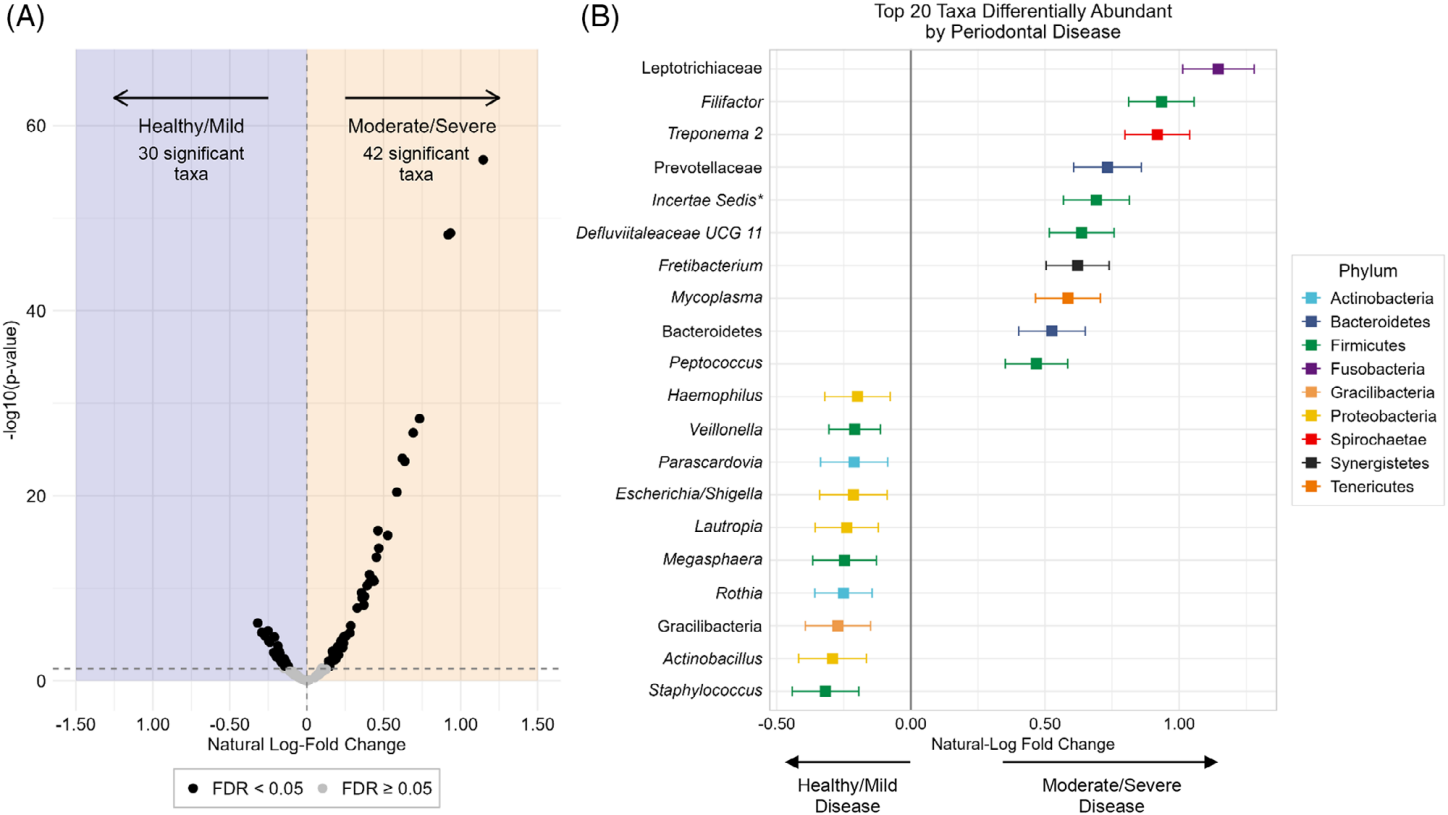
Differential abundance of salivary microbiota by periodontal disease status (*n* = 5037; NHANES 2009–2010, 2011–2012). Italicized taxa indicate genera. *Incertae Sedis indicates a microbe of the phylum Firmicutes from the Clostridiales Family XIII bacterium. The genus and species of this microbe were not identified in the taxonomic dataset in NHANES. FDR, false discovery rate; (F), family as the lowest taxonomic rank available; (P), phylum as the lowest taxonomic rank available. Plot (A) is a volcano plot visualizing salivary taxa that are significantly differentially abundant by periodontal disease status. Plot (B) is a log‐fold change plot which depicts the log‐fold change values and their 95% confidence intervals of the top 10 most differentially abundant taxa in periodontal disease and the top 10 most differentially abundant taxa in periodontal health.

Out of 121 available microbial taxa (most at genus‐level), 72 taxa were differentially abundant by periodontal disease (Figure 5A; FDR *q *< 0.05). From among these taxa, 42 were identified as enriched in moderate/severe periodontal disease (including Leptotrichiaceae [family], *Filifactor*, and *Treponema 2*) while 30 taxa were enriched in healthy/mild periodontal disease (including *Staphylococcus*, *Actinobacillus*, and Gracilibacteria [phylum]; Figure 5B). See Table S8 in the online *Journal of Periodontology* for a summarization across all available taxa.

## 4 DISCUSSION

In a nationally representative sample of US adults, we observed that baseline salivary microbiota predicted CMD mortality. An increased ratio of *Treponema* to *Corynebacterium* abundances quantified by MIP, an indicator of dysbiotic shifts in the oral microbiota commonly observed in periodontal disease, was associated with an elevated risk of CMD mortality. In contrast, neither salivary β‐diversity nor α‐diversity was predictive of CMD mortality. We did not observe an association between clinical periodontal disease and CMD mortality.

MIP was developed as a simple, microbiome‐based metric of periodontal disease in a community‐based cohort of young and middle‐aged adults without diabetes and with predominantly good oral health.^7^ MIP measured in both saliva and subgingival plaque was positively associated with oral microbial diversity and periodontal disease measures, including attachment loss, probing depth, and bleeding on probing.^7^ Moreover, MIP was associated with increased fasting insulin and insulin resistance–biomarkers of early CMD development.^7^ It is notable that in our current analysis, MIP was strongly related to CMD mortality, while neither a single taxon nor any commonly used measure of microbial community diversity was predictive of CMD mortality. This finding supports the notion that broad shifts in microbial consortia specifically related to periodontal disease may also influence cardiometabolic outcomes.^4^, ^5^, ^7^, ^40^

We view three possibilities for the role of periodontal disease in the relationships reported presently. First, it is possible that periodontal disease itself promotes microbial shifts of the biofilm,^3^ and thereby periodontal disease would be a confounder of the association. Alternatively, periodontal disease could influence mortality through the generation of subgingival dysbiosis coupled with inflammatory periodontal disease activity,^40^ which would frame oral microbiota as a mediator in the association between periodontitis and mortality. However, in models with mutual adjustment for both MIP and periodontal status, associations with mortality were not changed, such that MIP was strongly associated with CMD mortality, while associations between periodontal status and mortality were not statistically significant. The concurrent assessment of both microbiota and periodontal parameters only at baseline prevented us from better understanding the role of microbiota versus periodontal status. And a third scenario is that periodontal status modifies the microbiota‐mortality association. We hypothesized that MIP would show stronger associations with CMD mortality among participants with severe periodontitis compared with those with mild or no periodontitis because of (i) the evidence of local disease activity, and (ii) the enhanced potential for bacteremia in periodontitis. However, we observed the opposite in which MIP was most strongly associated with increased CMD mortality risks in individuals without periodontitis or with mild periodontitis. This could be a chance finding or it may suggest that early dysbiotic changes to the subgingival biofilm could influence CMD mortality before the onset of overt periodontal disease and risk above the threshold of MIP present in overt periodontitis might not confer additional risk. Future studies which utilize longitudinal oral microbiome and dental exam data collection are needed to better clarify the role of periodontal disease pertaining to oral microbiota as a predictor of mortality.

A key finding in our study is the demonstration that salivary biospecimens may be sensitive enough to capture subgingival microbial shifts predictive of CMD risk in a population‐based setting. This is important because saliva contains a mixture of microbiota from various surfaces within the oral cavity and the upper airway.^41^ If salivary MIP is a reproducible biomarker of microbial shifts toward subgingival dysbiosis, which contributes to periodontal disease, and is hypothesized to play a role in systemic disease, it could serve as a useful tool for detecting individuals at high risk of periodontal disease and/or CMD mortality. Nevertheless, our current results cannot prove causality, and validation of these findings in other population‐based cohorts is needed.

Our observation that increased salivary α‐diversity was associated with decreased all‐cause mortality risk is consistent with recent publications in NHANES.^14^, ^15^, ^16^ In contrast to previous studies which explored mortality subtypes,^15^, ^16^ our current findings do not show significant relationships between α‐diversity and CMD mortality. However, for CMD mortality, parameter estimates for select metrics including Shannon Diversity and Inverse Simpson indices—although consistent in direction and magnitude with prior studies—were not statistically significant in our analysis due to a notably smaller sample size.

Surprisingly, while we observed an inverse association between salivary α‐diversity and mortality, we found that higher levels of α‐diversity were associated with greater prevalence of periodontitis, despite previous studies linking periodontal disease to adverse cardiometabolic outcomes.^42^, ^43^ These seemingly contradictory findings are difficult to reconcile and could be the result of chance findings. Alternatively, it is possible that the non‐specific nature of diversity measures might explain this finding, as microbial diversity metrics encompass all salivary microbes, both commensals and pathobionts.^23^ Specifically, high α‐diversity in the context of periodontitis may signify a pro‐inflammatory state, which drives enrichment with a number of inflammophilic microbial taxa.^40^ In contrast, it is possible that high α‐diversity can emerge in the context of a healthy microbiota in which many health‐promoting taxa flourish. This is consistent with hypotheses related to the gut microbiome, where higher microbial diversity is consistently attributed to better systemic health outcomes.^44^ Lastly, the oral microbiome is a complex dynamic milieu, constantly subjected to environmental disturbances including dietary patterns, oral hygiene, medications, smoking, and cardiometabolic risk factors, which influence microbial diversity and composition within the oral cavity.^45^, ^46^

Several biological mechanisms may explain our findings related to MIP and CMD mortality. A leading hypothesis states that keystone pathogens of the subgingival biofilm, such as *Porphyromonas gingivalis* and *Aggregatibacter actinomycetemcomitans*, are posited to modulate complement system activation and other host immune responses, exacerbating gingival inflammation and enabling an expansion of periodontitis‐associated inflammophilic‐pathobionts.^47^, ^48^ Periodontal dysbiosis might diminish the abundance of beneficial microbiota, such as nitrate‐reducing microbes (including several *Corynebacterium* spp.), which are linked to nitric oxide production, lower insulin resistance, and anti‐inflammatory activity.^45^, ^49^ And lastly, oral virulence factors such as lipopolysaccharides and toxic gingipains and select strain‐specific microbiota can migrate through the bloodstream and localize systemically.^4^ Through oxidative stress and hyperinflammatory mechanisms, virulence factors are thought to amplify pancreatic insulin secretion, reduce the uptake of glucose in adipose and muscular tissues, and impair hepatic glucose metabolism, collectively contributing to potential downstream CMD dysfunction leading to CMD mortality.^4^, ^45^ Future research should investigate the functional capacities of microbes to identify oral microbial signatures and the biological processes underlying mortality associations.

Our study has several strengths. First, we used data from a large and demographically diverse cohort representative of the US adult population. Second, the prospective study design enabled clear temporal ordering, enhancing the interpretation of findings. Third, we evaluated the utility of an a priori microbiome‐based index of periodontal dysbiosis in predicting CMD mortality in a national cohort. Fourth, we incorporated both salivary microbiota data and clinical periodontal parameters to compare how well each set of measures predicted mortality. Lastly, we adjusted for important confounders including smoking, physical activity, and diet quality in our multivariable models.

There are also notable limitations. First, analyses were statistically underpowered due to a small number of cause‐specific mortality events. Future studies should consider assessing the relationship between oral microbiota and mortality outcomes in larger, older adult cohorts with higher mortality rates. Second, we were unable to examine changes in oral microbiota or cardiometabolic risk factors in the context of CMD mortality. Third, saliva may not fully capture the microbial diversity and composition of the subgingival biofilm, which is most relevant to periodontal disease. Finally, information on important health behaviors and healthcare, such as oral hygiene habits, prior periodontal treatments, and medication use, exhibited extensive missingness in NHANES. These unmeasured covariates may have led to an overestimation of the observed associations.

## 5 CONCLUSIONS

In this nationwide sample of US adults, higher levels of salivary *Treponema* to *Corynebacterium* predicted an increased risk of CMD mortality. If salivary MIP is a reproducible biomarker of periodontal disease and mortality risk, it could serve as a useful tool for detecting individuals for whom periodontal interventions might reduce CMD mortality risk. Future studies are needed to reproduce these findings and test whether interventions to improve the MIP decrease CMD risk.

## AUTHOR CONTRIBUTIONS

**Hamdi S. Adam** and **Ryan T. Demmer**: Study concept development. **Hamdi S. Adam**: Data preparation and analysis; preparing original manuscript drafts and manuscript revisions. **Weihua Guan, James S. Pankow**, and **Ryan T. Demmer**: Assisted in data analysis and manuscript revisions. **Abigail J. Johnson, Sanaz Sedaghat**, and **Charlene Goh**: Assisted in manuscript revisions.

## CONFLICT OF INTEREST STATEMENT

The authors declare no conflicts of interest.

## Supporting information



Supporting Information

Supporting Information

Supporting Information

Supporting Information

Supporting Information

Supporting Information

Supporting Information

Supporting Information

Supporting Information

Supporting Information

Supporting Information

Supporting Information

Supporting Information

Supporting Information

Supporting Information

Supporting Information

## Footnotes

* PCP2, Hu‐Friedy, Chicago, IL.
