# Supplementary material for: Salivary microbiota and clinical periodontal measures predicting cardiometabolic disease mortality: A nationwide survey
Source: J Periodontol. 2025 Oct 10;97(3):552–68. doi: 10.1002/jper.11395 (PMC12934248; doi:10.1002/jper.11395)
Supplement: Supplementary file 1 — Supporting Information [file JPER-97-552-s007.docx]

**Supplemental Figure 1**: Participant Flow Diagram (NHANES 2009-2010, 2011-2012)

Participants from the 2009-2010 & 2011-2012 survey cycles

N=20,293

(N=10,537 in 2009-2010;

N=9,756 in 2011-2012)

Adults ≥18 years, not pregnant

n=12,266

Exclusions: n=8,027

*(n=7,902 age <18;*

*n=125 pregnant)*

Complete saliva & dental examination data (age 30-69 years)

n=5,264

Exclusions: n=7,002

*(n=3,848 who did not provide saliva samples, or were ineligible [age >69 years];*

*n=278 with insufficient salivary α-diversity data at 10,000 sequence reads per sample threshold;*

*n=2,876 missing periodontal examination data or were ineligible for periodontal exam [age<30 years])*

**Final Sample:**

**Eligible for mortality data linkage**

**n=5,037**

Exclusions: n=227

*(n=9 ineligible for mortality data linkage;*

*n=218 missing key covariables [age, gender, race/ethnicity, education, income, smoking history])*
