# Supplementary material for: Salivary microbiota and clinical periodontal measures predicting cardiometabolic disease mortality: A nationwide survey
Source: J Periodontol. 2025 Oct 10;97(3):552–68. doi: 10.1002/jper.11395 (PMC12934248; doi:10.1002/jper.11395)
Supplement: Supplementary file 2 — Supporting Information [file JPER-97-552-s013.docx]

**Supplemental Figure 2**: Principal Coordinate Analysis of Non-Compositional Salivary β-Diversity Metrics (n=5,037; NHANES 2009-2010, 2011-2012)

*Unweighted UniFrac*

**A**

**B**


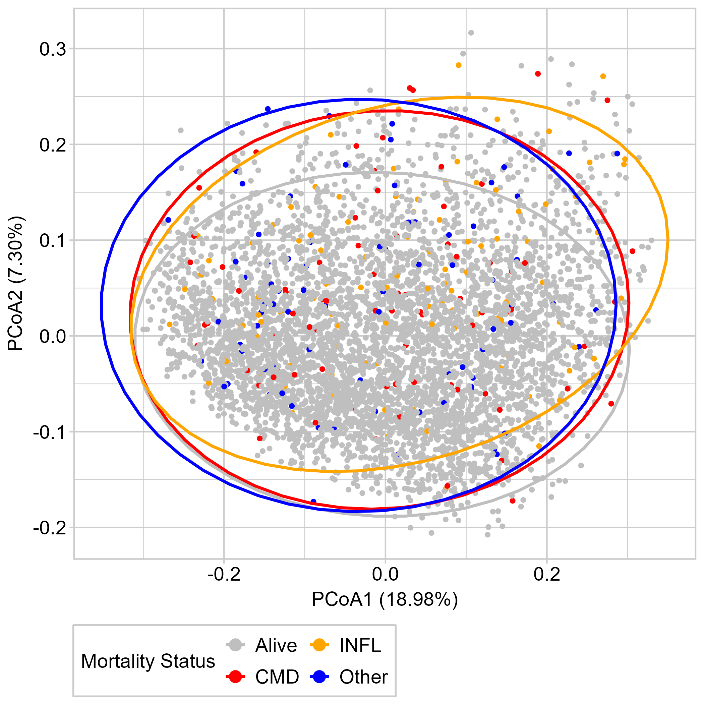


PERMANOVA

*p*-value = 0.95


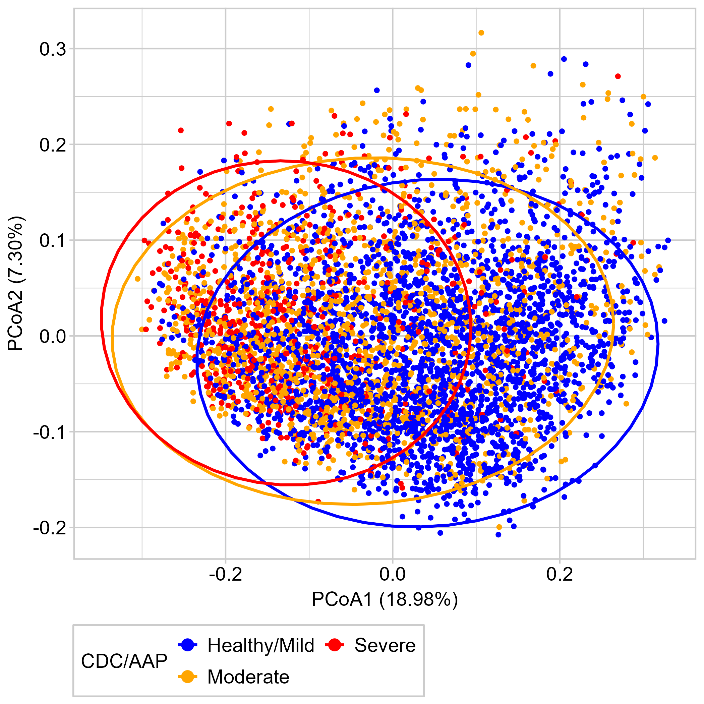


PERMANOVA

*p*-value = 0.97

*Weighted UniFrac*

**C**

**D**


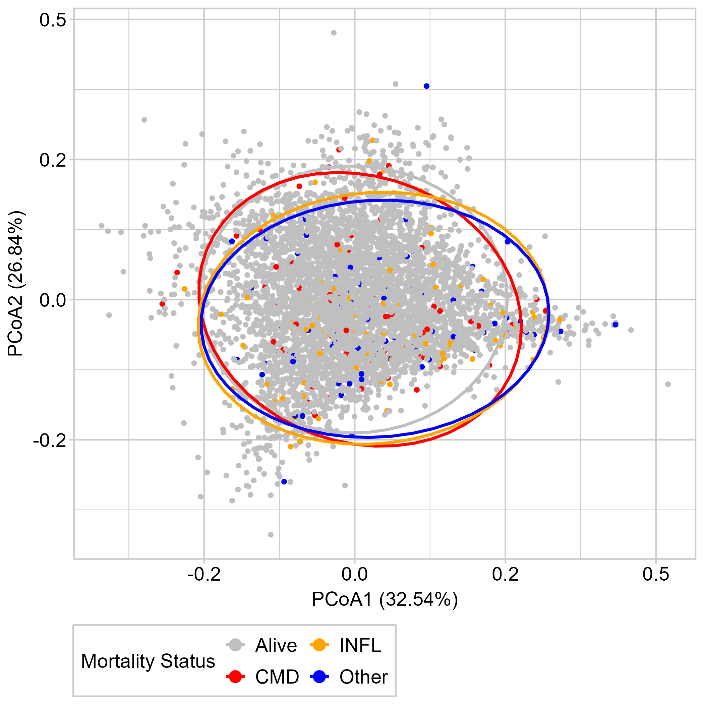


PERMANOVA

*p*-value = 0.70


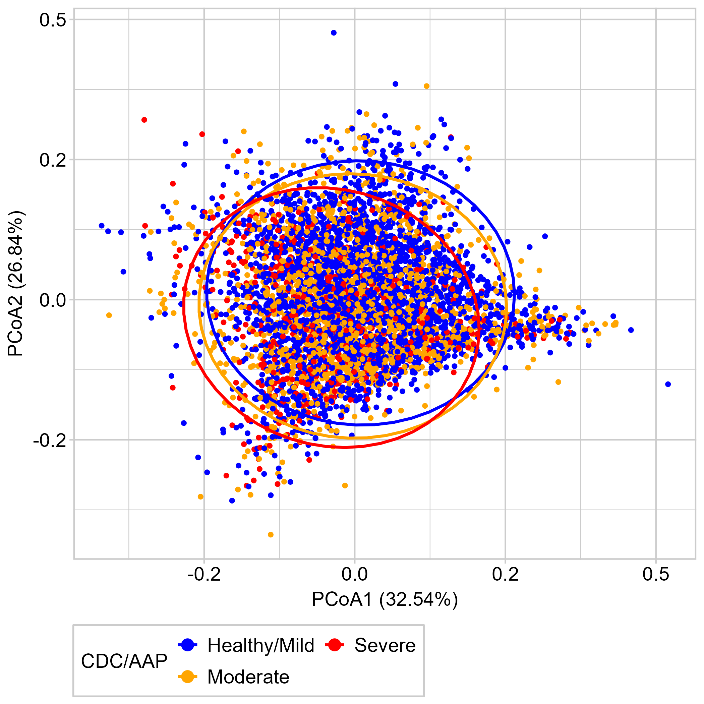


PERMANOVA

*p*-value = 0.35

*Bray-Curtis Dissimilarity*

*
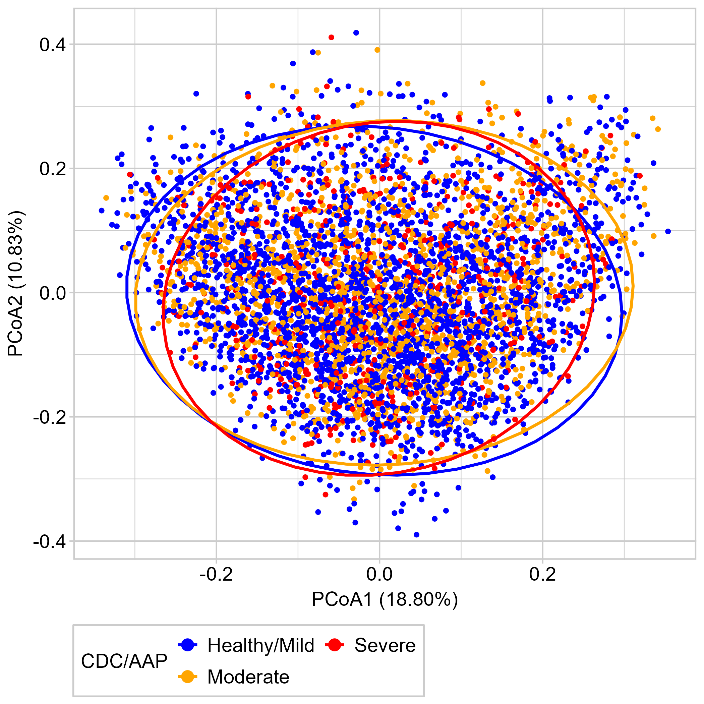
*

PERMANOVA

*p*-value = 0.69

PERMANOVA

*p*-value = 0.64

**E**

**F**

*
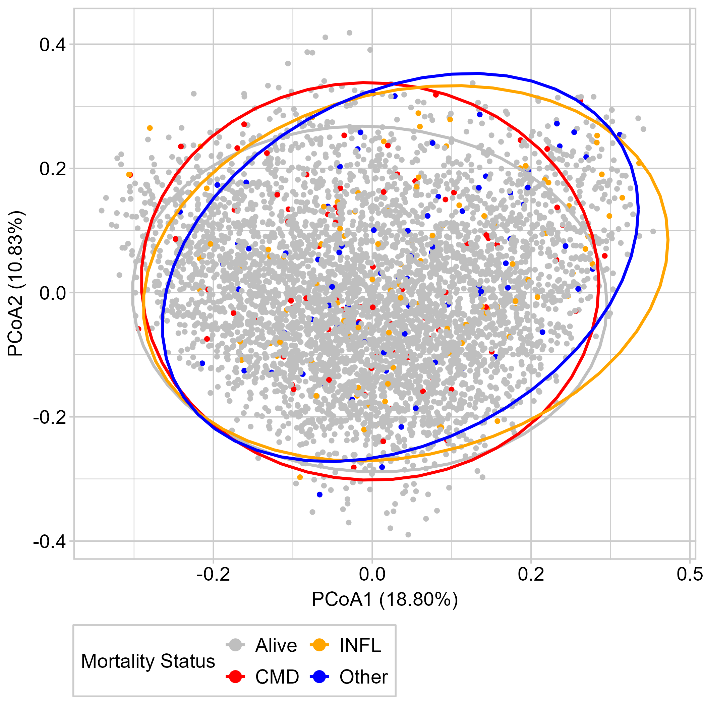
*

β-diversity distance matrices were generated by NHANES using the 10,000 sequence reads per sample rarefaction threshold. PERMANOVA p-values were generated through 999 permutations.

PCoA1: Principal Coordinate Analysis First Axis; PCoA2: Principal Coordinate Analysis Second Axis; CDC/AAP: Centers for Disease Control and Prevention/American Academy of Periodontology classification system; PERMANOVA: Permutational Multivariate Analysis of Variance; CMD = cardiometabolic disease mortality; INFL = inflammatory disease mortality
