# Supplementary material for: Salivary microbiota and clinical periodontal measures predicting cardiometabolic disease mortality: A nationwide survey
Source: J Periodontol. 2025 Oct 10;97(3):552–68. doi: 10.1002/jper.11395 (PMC12934248; doi:10.1002/jper.11395)
Supplement: Supplementary file 3 — Supporting Information [file JPER-97-552-s008.docx]

**Supplemental Figure 3**: Distributions of Salivary α-Diversity Metrics (n=5,037; NHANES 2009-2010, 2011-2012)


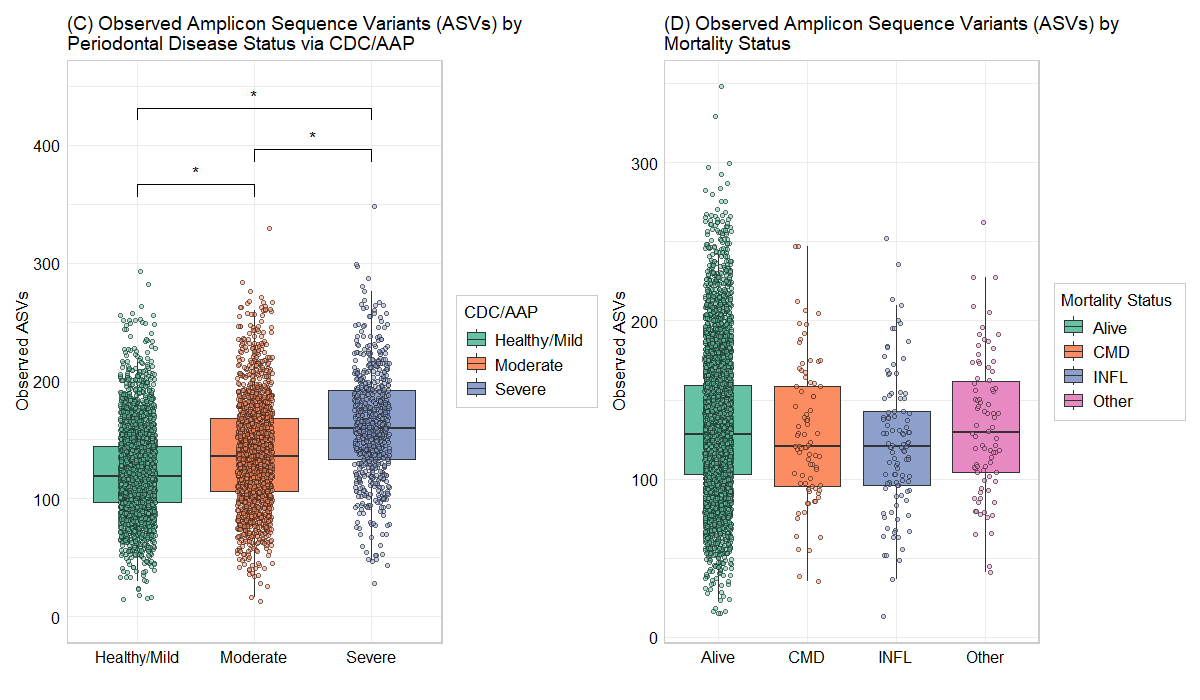
*Observed ASVs*

ANOVA *p*-value: 0.06

**ANOVA *p*-value: < 0.01**

**B**

**A**


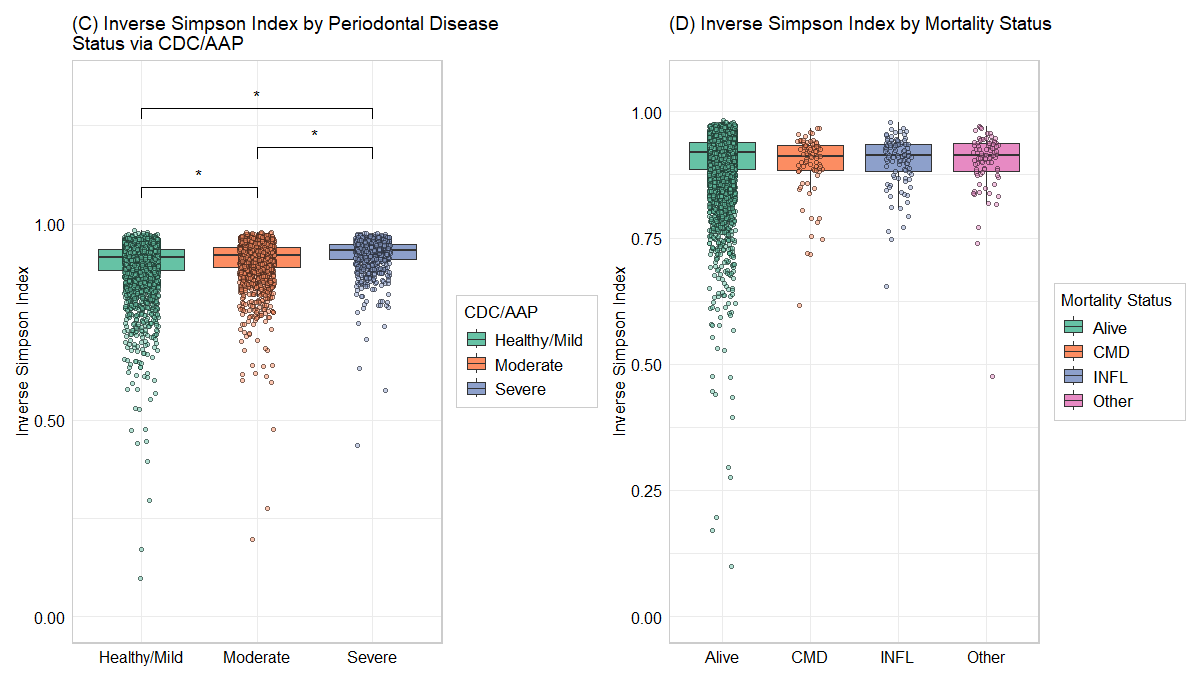
*Inverse Simpson Index*

**D**

**C**

**Kruskal-Wallis *p*-value: < 0.01**

Kruskal-Wallis *p*-value: 0.19

*Faith’s Phylogenetic Diversity*


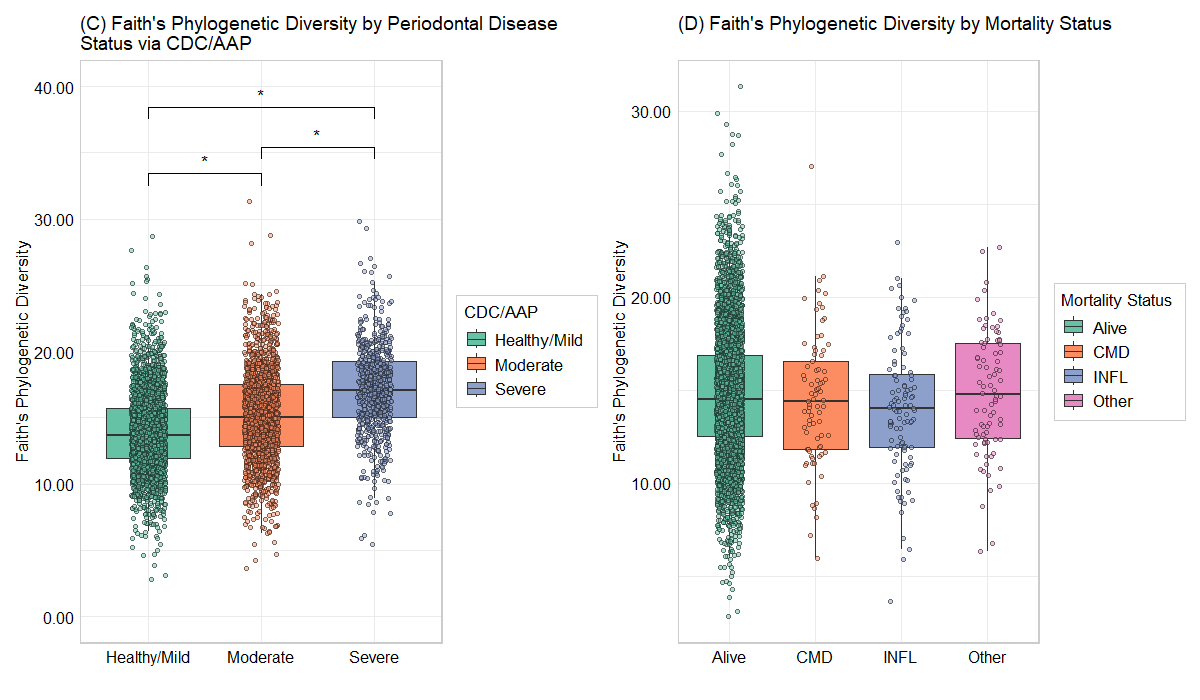


ANOVA *p*-value: 0.12

**ANOVA *p*-value: < 0.01**

**E**

**F**

Parametric ANOVA and pairwise t-tests were used to assess observed ASVs. Non-parametric Kruskal-Wallis and pairwise Mann-Whitney U tests were used to assess Shannon Diversity and Inverse Simpson Index. Statistical significance (p-value <0.05) is represented with asterisks (for pairwise comparisons) and bold font (global test). CDC/AAP: Centers for Disease Control and Prevention/American Academy of Periodontology classification system; ANOVA: Analysis of Variance; CMD: Cardiometabolic Disease Mortality; INFL: Chronic Inflammatory Disease Mortality. *p*-value <0.05 indicated statistical significance (via asterisks).
