# Supplementary material for: Salivary microbiota and clinical periodontal measures predicting cardiometabolic disease mortality: A nationwide survey
Source: J Periodontol. 2025 Oct 10;97(3):552–68. doi: 10.1002/jper.11395 (PMC12934248; doi:10.1002/jper.11395)
Supplement: Supplementary file 4 — Supporting Information [file JPER-97-552-s014.docx]

**Supplemental Material 1**: Procedures for Salivary Microbiota Data Generation (NHANES; 2009-2010, 2011-2012)

Microbiome Data Generation

Saliva samples were collected by NHANES staff in MECs from participants ≤69 years old during the 2009-2010 and 2011-2012 survey cycles with a 10 mL mouthwash rinse for 30 seconds.^1-3^ Deidentified saliva samples were transported to the Knight Laboratory at the University of California, San Diego for 16S rRNA gene sequencing, computational processing, and microbiome data generation.^1^ DNA extracts from salivary bacteria underwent polymerase chain reactions targeting the V4 hypervariable region of the 16S rRNA gene using V4 515F-806R primers ~390 base pair (bp) amplicons.^1^ DNA amplicons were sequenced by an Illumina HiSeq 2500 in pairs of 125 bp, demultiplexed through the *QIIME* bioinformatic pipeline,^4^ and subsequently processed with the Divisive Amplicon Denoising Algorithm 2 (*DADA2*)^5^ to generate an amplicon sequence variance table.^1^ Bacterial taxonomies were assigned using the SILVA reference database (version 123).^1^

Microbiome Diversity Metrics

β-diversity metrics, which quantify microbial similarities and differences between salivary microbiome samples, were produced in *QIIME* through ten iterations of without-replacement rarefaction at 10,000 sequence reads/sample and stored as publicly available distance matrices.^1^ These non-compositional metrics (meaning they do not correct for microbial compositionality) include weighted UniFrac (incorporates both bacterial phylogenies and relative abundances), unweighted UniFrac (considers only bacterial phylogenies), and Bray-Curtis dissimilarity (uses only bacterial relative abundances).^6^ We additionally calculated Aitchison Distance, a compositional β-diversity metric that corrects for microbial compositionality, by incorporating ASV tables into PCoA.^7^ α-diversity metrics, which measure microbial diversity within salivary microbiome samples, were produced by *QIIME* and similarly rarefied at 2,000, 4,000, 6,000, 8,000, and 10,000 sequence reads/sample thresholds without replacement.^1^ α-diversity metrics included the observed number of amplicon sequence variants (observed ASVs, a measure of bacterial species richness), Shannon diversity index and Inverse Simpson index (both of which estimate species evenness and richness), and Faith’s phylogenetic diversity (incorporates bacterial phylogenies).^6^ β-diversity metric data were stored by NHANES as distance matrices while α-diversity metric data were stored as data tables, both of which are publicly available data files.^1^

For β-diversities, we employed principal coordinates analyses (PCoA) and used the first and second PCoA axes to separately quantify salivary microbial dissimilarity for each β-diversity metric. First axis PCoA β-diversities were standardized into z-scores and categorized into tertiles. We additionally calculated mean salivary α-diversities across rarefaction iterations at the 10,000 sequence reads/sample threshold for each α-diversity metric, according to rarefaction data from NHANES.^2^ Mean salivary α-diversities were also standardized into z-scores and categorized into tertiles.

Differential Abundance Analysis

We conducted differential abundance analyses of microbial taxa using the 2^nd^ edition of the Analysis of Compositions of Microbiomes with Bias Correction function (*ANCOMBC 2*) from the *ancombc* package (v.2.6.1) in R.^8^ Using taxonomic data publicly available from NHANES, we tested for differential abundances of microbial taxa across periodontal disease status (moderate/severe disease vs healthy/mild).^8^ *ANCOMBC 2* identified differentially abundant taxa through a false discovery rate (FDR) multiple comparisons correction of q<0.05. The direction and strength of association between taxa and periodontal disease is quantified by natural log-fold changes.^8^ That is, positive log-fold change values indicated taxa that were enriched in more moderate to severe forms of periodontal disease while negative log-fold change values indicated taxa that were enriched in periodontal health.

In our differential abundance analyses, we i) used the salivary taxonomic ASV count table and participant metadata as input; ii) set a significance threshold of 0.05; iii) excluded microbial taxa that were present in <10% of saliva samples; iv) excluded saliva samples with <1000 sequence reads; and v) used FDR to account for multiple comparisons. We adjusted for the following covariables: age, sex, race/ethnicity, education, annual income, smoking history, BMI, and diet quality. Differentially abundance analyses were conducted at the genera level. In the ASV tables provided by NHANES, some genus-level taxonomies were not available for select microbes. For microbes with missing genus taxonomies, we assigned the lowest available taxonomic rank available (i.e. family, order, class, phylum).

**References**

1. Centers for Disease Control and Prevention. National Health and Nutrition Examination Survey - 2009-2010 and 2011-2012 oral microbiome data documentation. 2022:1-12.

2. Vogtmann E, Chaturvedi AK, Blaser MJ, et al. Representative oral microbiome data for the US population: the National Health and Nutrition Examination Survey. *Lancet Microbe.* 2023;4(2):e60-e61.

3. Gillison ML, Broutian T, Pickard RKL, et al. Prevalence of Oral HPV Infection in the United States, 2009-2010. *JAMA.* 2012;307(7):693.

4. Caporaso JG, Kuczynski J, Stombaugh J, et al. QIIME allows analysis of high-throughput community sequencing data. *Nature Methods.* 2010;7(5):335-336.

5. Callahan BJ, McMurdie PJ, Rosen MJ, Han AW, Johnson AJA, Holmes SP. DADA2: High-resolution sample inference from Illumina amplicon data. *Nature Methods.* 2016;13(7):581-583.

6. Emmons AL, Chill SS. Microbiome analysis with QIIME2. 2023:Online bioinformatics training and education program.

7. Martino C, Morton JT, Marotz CA, et al. A Novel Sparse Compositional Technique Reveals Microbial Perturbations. *mSystems.* 2019;4(1).

8. Lin H, Peddada SD. Multigroup analysis of compositions of microbiomes with covariate adjustments and repeated measures. *Nature Methods.* 2024;21(1):83-91.
