# Supplementary material for: Salivary microbiota and clinical periodontal measures predicting cardiometabolic disease mortality: A nationwide survey
Source: J Periodontol. 2025 Oct 10;97(3):552–68. doi: 10.1002/jper.11395 (PMC12934248; doi:10.1002/jper.11395)
Supplement: Supplementary file 5 — Supporting Information [file JPER-97-552-s010.docx]

**Supplemental Material 2**: Full-Mouth Periodontal Examination Procedures (NHANES; 2009-2010, 2011-2012)

Full-mouth periodontal examinations were conducted in MECs to adult participants 30 and older with at least one natural tooth present (excluded edentulous participants), who also do not have medical conditions that need antibiotics prior to full-mouth examination during the 2009-2010 and 2011-2012 NHANES survey cycles.^1^ This selection criteria was likely applied by NHANES because periodontal disease, particularly moderate to severe periodontitis, is generally more prevalent among adults age 30 and older.^1^

Trained and calibrated dental examiners used periodontal probes* to measure gingival recessions (millimeters from free gingival margin to cemento-enamal junction) and periodontal probing depths (millimeters from free gingival margin to bottom of sulcus [for healthy sites] or periodontal pocket [for diseased sites]) at six sites per tooth (mesiobuccal, midbuccal, distobuccal, mesiolingual, midlingual, distolingual) across all available teeth, with exception to third molars.^1^ Examiners calculated clinical attachment loss by taking the difference between periodontal probing depths and gingival recession values.^1^

Our secondary exposure for this study are measures of clinical periodontal disease. We calculated mean interproximal periodontal probing depth (I-PPD) and interproximal clinical attachment loss (I-CAL) from mesial and distal sites across all available teeth for each participant. Unlike full-mouth measures which include midbuccal and midlingual sites that are more susceptible to traumatic gingival recessions, interproximal sites were selected to better capture gingival recessions associated with periodontal disease.^2^ Mean I-PPD and I-CAL were operationalized continuously (mm), standardized into z-scores, and into tertiles. We then incorporated probing depth and attachment loss measures in characterizing periodontal disease according to the Centers for Disease Control/American Academy of Periodontology system (CDC/AAP), which defined periodontitis as *healthy, mild*, *moderate*, or *severe*.^1^ Note that we modified this categorization and combined *healthy* and *mild* periodontal disease into a single level for regression modeling due to small numbers of participants having mild disease. For this study, we considered I-PPD reflective of current periodontal tissue inflammation, I-CAL to characterize historic periodontal tissue destruction, and CDC/AAP as an indicator of both.^1,2^

*See* ***Supplemental Table 1*** for the correlations between continuous β-diversity PCoA axes, α-diversity metrics, periodontal measures, and Microbial Indicator of Periodontitis (MIP).

**References**

1. Eke PI, Thornton-Evans GO, Wei L, Borgnakke WS, Dye BA, Genco RJ. Periodontitis in US adults: National Health and Nutrition Examination Survey 2009-2014. *J Am Dent Assoc.* 2018;149(7):576-588.e576.

2. Adam HS, Molinsky R, Bohn B, et al. Clinical attachment loss is cross‐sectionally associated with elevated glucose among adults without diabetes. *J Clin Periodontol.* 2024.

*PCP2 Hu-Friedy
