# Supplementary material for: Salivary microbiota and clinical periodontal measures predicting cardiometabolic disease mortality: A nationwide survey
Source: J Periodontol. 2025 Oct 10;97(3):552–68. doi: 10.1002/jper.11395 (PMC12934248; doi:10.1002/jper.11395)
Supplement: Supplementary file 6 — Supporting Information [file JPER-97-552-s001.docx]

**Supplemental Material 3**: Ascertainment and Operationalization of Baseline Covariables (NHANES; 2009-2010, 2011-2012)

Baseline sociodemographic variables ascertained from self-reported at-home interviews included age, gender (male/female), race/ethnicity (Mexican American, Other Hispanic, Non-Hispanic White, Non-Hispanic Black, Other/non-Hispanic Multiracial), education (some high school, high school diploma/GED, some college/associates degree, college graduate or higher), and annual family income (<$20,000; $20,000-$75,000; >$75,000). Body mass index (BMI) from physical exams at MECs was operationalized continuously (kg/m^2^) and categorically per CDC guidelines (underweight/normal weight: <25 kg/m^2^; overweight: 25-30 kg/m^2^; obese: 30 kg/m^2^).^1^ Using the *dietaryindex* R package,^2^ diet quality was assessed with the Alternative Healthy Eating Index (AHEI), which incorporated eleven dietary items from the Day 1 NHANES 24-hour food recall.^3^ Intake of the following dietary components are used in AHEI: intake of fruits, vegetables, nuts, red meat, sugar-sweetened beverages, omega-3 fatty acids, polyunsaturated fats, trans fats, alcohol, and whole grains and refined grains.^4^ The possible range of AHEI scores is from 0 to 110. Weekly physical activity level was estimated via the metabolic equivalent of tasks score (MET) and categorized using the Physical Activity Guidelines for Americans: <500 MET min/week, 500-1000 MET min/week, >1000 MET min/week.^5-7^ As previously done in NHANES, smoking history was derived from two smoking questionnaire items during the at-home survey: whether a participant smoked ≥100 cigarettes during their lifetime and whether they are currently smoking.^8^ Smoking history was categorized as follows: never smoker = did not smoke at least 100 cigarettes in lifetime; former smoker = smoked at least 100 cigarettes in lifetime and currently are nonsmokers; current smokers = smoked at least 100 cigarettes in lifetime and report currently smoking every day or some days.^8^ Mean systolic and diastolic blood pressures were calculated from three consecutive readings at MECs. Prevalent hypertension was determined by systolic blood pressure ≥130mmHg, diastolic blood pressure ≥80mmHg, or self-reported anti-hypertensive medication use, per American College of Cardiology/American Heart Association guidelines.^9^ Total cholesterol^*^ and glycated hemoglobin A1c (HbA1c)^†^ were measured and analyzed during MEC visits from non-fasting blood samples. Prediabetes was defined by HbA1c of 5.7-6.5% or self-reported prediabetes diagnosis, while diabetes was classified as HbA1c ≥6.5% or self-reported diabetes diagnosis.^10^ High cholesterol was determined by total cholesterol ≥200mg/dL.^11^ Prevalent cardiovascular disease or kidney disease was defined via self-reported diagnosis of congestive heart failure, coronary heart disease, angina pectoris, heart attack, stroke, or weak/failing kidneys.

**References**

1. McDowell MA, Hughes JP, Borrud LG. Health characteristics of U.S. adults by body mass index category: results from NHANES 1999–2002. *Public Health Rep.* 2006;121(1):67-73.

2. Zhan JJ, Hodge RA, Dunlop AL, et al. Dietaryindex: A User-Friendly and Versatile R Package for Standardizing Dietary Pattern Analysis in Epidemiological and Clinical Studies. In: Cold Spring Harbor Laboratory; 2023.

3. Wang DD, Leung CW, Li Y, et al. Trends in dietary quality among adults in the United States, 1999 through 2010. *JAMA Intern Med.* 2014;174(10):1587.

4. Adam HS, Molinsky R, Bohn B, et al. Clinical attachment loss is cross‐sectionally associated with elevated glucose among adults without diabetes. *J Clin Periodontol.* 2024.

5. Ainsworth BE, Haskell WL, Whitt MC, et al. Compendium of physical activities: an update of activity codes and MET intensities. *Medicine & Science in Sports & Exercise.* 2000;32(9):S498-S516.

6. Du Y, Liu B, Sun Y, Snetselaar LG, Wallace RB, Bao W. Trends in adherence to the Physical Activity Guidelines for Americans for aerobic activity and time spent on sedentary behavior among US adults, 2007 to 2016. *JAMA Netw Open.* 2019;2(7):e197597.

7. United States Department of Health and Human Services. 2008 Physical Activity Guidelines for Americans. In*.* Vol 2023. 1st ed. Washington D.C.2008:5.

8. Zhu D, Zhao G, Wang X. Association of smoking and smoking cessation with overall and cause-specific mortality. *Am J Prev Med.* 2021;60(4):504-512.

9. Whelton PK, Carey RM, Aronow WS, et al. 2017 ACC/AHA/AAPA/ABC/ACPM/AGS/APhA/ASH/ASPC/NMA/PCNA Guideline for the prevention, detection, evaluation, and management of high blood pressure in adults: a report of the American College of Cardiology/American Heart Association task force on clinical practice guidelines. *Hypertension.* 2018;71(6):e13-e115.

10. ElSayed NA, Aleppo G, Aroda VR, et al. 2. classification and diagnosis of diabetes: Standards of Care in Diabetes--2023. *Diabetes Care.* 2023;46(Suppl 1):S19-s40.

11. Grundy SM, Stone NJ, Bailey AL, et al. 2018 AHA/ACC/AACVPR/AAPA/ABC/ACPM/ADA/AGS/APhA/ASPC/NLA/PCNA guideline on the management of blood cholesterol: a report of the American College of Cardiology/American Heart Association Task Force on Clinical Practice Guidelines. *Circulation.* 2019;139(25).

12. National Center for Health Statistics. 2009-2010 laboratory data - continuous NHANES. Centers for Disease Control and Prevention. National Health and Nutrition Examination Survey Web site. h<ttps://wwwn.cdc.gov/nchs/nhanes/search/datapage.aspx?Component=Laboratory&Cycle=2009-2010.> Accessed April 30, 2024.

13. National Center for Health Statistics. 2011-2012 laboratory data - continuous NHANES. Centers for Disease Control and Prevention. National Health and Nutrition Examination Survey Web site. <https://wwwn.cdc.gov/nchs/nhanes/Search/DataPage.aspx?Component=Laboratory&CycleBeginYear=2011>. Accessed April 30, 2024.

*Total blood cholesterol analyzed by a Roche Modular P Chemistry Analyzer.^12,13^

^†^HbA1c was analyzed by a G7 Glycohemoglobin Analyzer (Tosoh Medics, Inc., San Francisco, CA).^12,13^
