# Supplementary material for: Salivary microbiota and clinical periodontal measures predicting cardiometabolic disease mortality: A nationwide survey
Source: J Periodontol. 2025 Oct 10;97(3):552–68. doi: 10.1002/jper.11395 (PMC12934248; doi:10.1002/jper.11395)
Supplement: Supplementary file 7 — Supporting Information [file JPER-97-552-s009.docx]

**Supplemental Material 4**: Statistical Analysis Information (NHANES; 2009-2010, 2011-2012)

Principal Coordinates Analysis of β-diversity dissimilarity

We used the *ape* R package to conduct principal coordinates analysis (PCoA) to explore β-diversity dissimilarities across periodontal disease status (via Centers for Disease Control and Prevention/American Academy of Periodontology classification [CDC/AAP]) and mortality status.^1^ Four β-diversity metrics were used: unweighted UniFrac, weighted UniFrac, and Bray-Curtis dissimilarity (non-compositional metrics) and Aitchison’s distance (compositional). Using the *vegan* R package, we applied Permutational Multivariate Analysis of Variance (PERMANOVA) with 999 permutations to test for statistical differences across periodontal disease categories and mortality status in our PCoA explorations.^2^ PCoA plots using the first and second PCoA axes were generated using the *ggplot2* package in R.^3^

Exploration of α-diversity distributions

We examined α-diversity distributions across periodontal disease categories and mortality status using pairwise and global statistical tests. For observed amplicon sequencing variants (ASVs) and Faith’s phylogenetic diversity metrics, we used t-tests and analysis of variance (ANOVA) for parametric pairwise comparisons and global assessments of α-diversity distributions, respectively. As for Shannon index and Inverse Simpson index, we used Mann-Whitney U tests and Kurskal-Wallis tests for non-parametric pairwise comparisons and global assessments of α-diversity distributions, respectively. Box and whisker plots were generated using the *ggplot2* package in R.^3^

Normalized Relative Abundances

We computed normalized relative abundance of microbial phyla within each category/level of Shannon index, periodontal disease status, and mortality outcomes. Stacked bar charts were generated using the *ggplot2* package in R.^3^

Differential Abundance Analysis

We used the Analysis of Compositions of Microbiomes with Bias Correction 2 (ANCOMBC 2) bioinformatic procedure^4^ to test for differential abundances of microbial taxa across periodontal disease status (moderate/severe disease vs healthy/mild disease). Complete details of this approach are found in ***Supplemental Material 1***.

Output from ANCOM-BC2 was used to visualize differentially abundant taxa by periodontal disease status in a Manhattan plot, generated with the *ggplot2* package in R.^3^ The plot depicts all 121 taxa and their respective phyla plotted by their negative log-10 p-values. Taxa were determined significantly differentially abundant via false discovery rate (FDR) q-value of <0.05, which adjusted for multiple comparisons.

Similarly, the strength of association between the abundance of the top 20 differentially abundant microbial taxa and periodontal disease status was presented in a natural log-fold change chart, also generated with the ggplot2 package in R.^3^ Positive natural-log fold change values indicated taxa that were enriched with periodontal disease while negative natural-log fold change values indicated taxa that were enriched with periodontal health.

Imputation Approaches for Missing Covariable Observations

We conducted single-median imputations for continuous covariables with <5% missing observations (AHEI, BMI, HbA1c, total cholesterol, systolic and diastolic blood pressures), and categorized missing MET observations, which comprised >20% of the sample, as “*missing*”.

Proportional Hazards Modeling in Primary Analysis

We used the *survey* package in R to conduct survey-weighted multivariable Cox regression, which estimated hazard ratios and 95% confidence intervals (HR [95% CI]) of mortality outcomes across β-diversity PCoA axes (reference = Tertile 1), α-diversity (reference = Tertile 1), periodontal variables (reference = Tertile 1 or healthy/mild), and MIP (reference = Tertile 1). Mortality rates were also regressed by continuous β-diversity, α-diversity, I-CAL, I-PPD, and MIP (standardized as z-scores). The following covariable adjustments were used:

- Model 1: adjustment for survey cycle
- Model 2: Model 1 + age, gender, race/ethnicity, education, income
- Model 3: Model 2 + BMI, AHEI, physical activity, smoking
- Model 4: Model 3 + CDC/AAP (when exposure = MIP, first axis PCoA β-diversity, or α-diversity ) or Shannon Index (when exposure = periodontal measure)
- Model 5: Model 4 + HbA1c, systolic blood pressure, total cholesterol.

Hazard ratios and 95% confidence intervals of mortality outcomes were computed per 1-standard deviation of our exposure variables and across tertiles of our exposure variables in main regression results. A forest plot summarizing the relationship between standardized oral microbiota indices and mortality outcomes displays natural-log transformed HRs (95% CIs) to enhance interpretation of effect estimates was also constructed.

Robust-Variance Poisson Regression Modeling in Secondary Analysis

We used the *survey* package in R to conduct survey-weighted multivariable robust-variance Poisson regression to estimate prevalence ratios and 95% confidence intervals (PR [95% CI]) of moderate/severe periodontal disease (via CDC/AAP) across MIP (per 1-SD and by tertiles; reference = Tertile 1). The following covariable adjustments were used:

- Model 1: adjustment for survey cycle
- Model 2: Model 1 + age, gender, race/ethnicity, education, income
- Model 3: Model 2 + BMI, AHEI, physical activity, smoking
- Model 4: Model 3 + Shannon Index
- Model 5: Model 4 + HbA1c, systolic blood pressure, total cholesterol.

Proportional Hazards Modeling in Subgroup Analysis

We used the *survey* package in R to conduct survey-weighted multivariable Cox regression, which estimated hazard ratios and 95% confidence intervals (HR [95% CI]) of CMD and all-cause mortality across standardized MIP and Shannon diversity index. The following covariable adjustments were used:

- MIP models stratified by periodontal disease adjust for survey cycle, age, gender, race/ethnicity, education, income, body mass index, Alternative Healthy Eating Index, physical activity, smoking history, HbA1c, systolic blood pressure, and total cholesterol.
- Shannon diversity index models stratified by periodontal disease adjust for survey cycle, age, gender, race/ethnicity, education, income, body mass index, Alternative Healthy Eating Index, physical activity, smoking history, HbA1c, systolic blood pressure, and total cholesterol.
- MIP x periodontal disease interaction models adjust for survey cycle, age, gender, race/ethnicity, education, income, body mass index, Alternative Healthy Eating Index, physical activity, smoking history, HbA1c, systolic blood pressure, and total cholesterol.
- Shannon diversity index x periodontal disease interaction models adjust for survey cycle, age, gender, race/ethnicity, education, income, body mass index, Alternative Healthy Eating Index, physical activity, smoking history, HbA1c, systolic blood pressure, and total cholesterol.

Statistical Software

Organization of periodontal measures was done in PC-SAS version 9.4*. Data cleaning, manipulation, and analyses were completed in R version 4.3.2.^†^ All plots and data visualizations were created using the *ggplot2* R package.^3^ *p*-values <0.05 indicated statistical significance. Bonferroni adjustment was used in summary analysis to adjust for multiple comparisons. We assumed a Type 1 error rate of 0.05 with 33 statistical comparisons, generating a Bonferroni-adjusted significance threshold of 0.0025 (0.05 type 1 error rate ÷ 20 comparisons).

**References**

1. Paradis E, Schliep K. ape 5.0: an environment for modern phylogenetics and evolutionary analyses in R. *Bioinformatics.* 2019;35(3):526-528.

2. Oksanen J, Simpson G, Blanchet F, et al. vegan: community ecology package. 2024.

3. Wickham H. *ggplot2: elegant graphics for data analysis.* Springer-Verlag New York; 2016.

4. Lin H, Peddada SD. Multigroup analysis of compositions of microbiomes with covariate adjustments and repeated measures. *Nature Methods.* 2024;21(1):83-91.

*PC-SAS version 9.4, SAS Institute Inc., Cary, NC.

†R version 4.3.2, The R Project for Statistical Computing.
