# Supplementary material for: Salivary microbiota and clinical periodontal measures predicting cardiometabolic disease mortality: A nationwide survey
Source: J Periodontol. 2025 Oct 10;97(3):552–68. doi: 10.1002/jper.11395 (PMC12934248; doi:10.1002/jper.11395)
Supplement: Supplementary file 8 — Supporting Information [file JPER-97-552-s016.docx]

**Supplemental Table 1:** Pearson Correlation Coefficient Matrix of Oral Microbial Indices (n=5,037; NHANES 2009-2010, 2011-2012)

|  | **W-UF-A1** | **Unw-UF-A1** | **BC-A1** | **W-UF-A2** | **Unw-UF-A2** | **BC-A2** | **Obs. ASVs** | **Shannon** | **Inv. Simpson** | **Faith's** | **I-CAL** | **I-PPD** | **MIP** |
| --- | --- | --- | --- | --- | --- | --- | --- | --- | --- | --- | --- | --- | --- |
| **W-UF-A1** | 1 | 0.37 | 0 | 0 | 0.36 | 0.75 | -0.43 | -0.66 | -0.56 | -0.38 | -0.06 | -0.18 | -0.24 |
| **Unw-UF-A1** | 0.37 | 1 | 0.09 | 0.14 | 0 | 0.05 | -0.89 | -0.64 | -0.33 | -0.92 | -0.3 | -0.47 | -0.66 |
| **BC-A1** | 0 | 0.09 | 1 | -0.57 | 0.46 | 0 | -0.1 | 0.24 | 0.44 | -0.1 | 0.04 | -0.01 | 0.06 |
| **W-UF-A2** | 0 | 0.14 | -0.57 | 1 | -0.37 | -0.17 | -0.13 | -0.31 | -0.24 | -0.12 | -0.13 | -0.16 | -0.17 |
| **Unw-UF-A2** | 0.36 | 0 | 0.46 | -0.37 | 1 | 0.55 | -0.05 | -0.07 | -0.03 | -0.02 | 0.17 | 0.16 | 0.17 |
| **BC-A2** | 0.75 | 0.05 | 0 | -0.17 | 0.55 | 1 | -0.11 | -0.41 | -0.46 | -0.08 | 0.06 | 0.03 | 0.01 |
| **Obs. ASVs** | -0.43 | -0.89 | -0.1 | -0.13 | -0.05 | -0.11 | 1 | 0.72 | 0.39 | 0.96 | 0.24 | 0.41 | 0.48 |
| **Shannon** | -0.66 | -0.64 | 0.24 | -0.31 | -0.07 | -0.41 | 0.72 | 1 | 0.84 | 0.69 | 0.18 | 0.3 | 0.35 |
| **Inv. Simpson** | -0.56 | -0.33 | 0.44 | -0.24 | -0.03 | -0.46 | 0.39 | 0.84 | 1 | 0.37 | 0.1 | 0.15 | 0.19 |
| **Faith's** | -0.38 | -0.92 | -0.1 | -0.12 | -0.02 | -0.08 | 0.96 | 0.69 | 0.37 | 1 | 0.25 | 0.42 | 0.53 |
| **I-CAL** | -0.06 | -0.3 | 0.04 | -0.13 | 0.17 | 0.06 | 0.24 | 0.18 | 0.1 | 0.25 | 1 | 0.69 | 0.24 |
| **I-PPD** | -0.18 | -0.47 | -0.01 | -0.16 | 0.16 | 0.03 | 0.41 | 0.3 | 0.15 | 0.42 | 0.69 | 1 | 0.36 |
| **MIP** | -0.24 | -0.66 | 0.06 | -0.17 | 0.17 | 0.01 | 0.48 | 0.35 | 0.19 | 0.53 | 0.24 | 0.36 | 1 |

Abbreviations: W-UF-A1 = Weighted UniFrac PCoA Axis 1; Unw-UF-A1 = Unweighted UniFrac PCoA Axis 1; BC-A1 = Bray-Curtis PCoA Axis 1; W-UF-A2 = Weighted UniFrac PCoA Axis 2; Unw-UF-A2 = Unweighted UniFrac PCoA Axis 2; BC-A2 = Bray-Curtis PCoA Axis 2; Obs. ASVs = Observed amplicon sequence variants; Shannon = Shannon Diversity Index; Inv. Simpson = Inverse Simpson; Faith’s = Faith’s Phylogenetic Diversity Index; I-CAL = interproximal clinical attachment loss; I-PPD = interproximal periodontal probing depth; PCoA = Principal Coordinates Analysis; MIP = Microbial Indicator of Periodontitis.
