# Supplementary material for: Salivary microbiota and clinical periodontal measures predicting cardiometabolic disease mortality: A nationwide survey
Source: J Periodontol. 2025 Oct 10;97(3):552–68. doi: 10.1002/jper.11395 (PMC12934248; doi:10.1002/jper.11395)
Supplement: Supplementary file 9 — Supporting Information [file JPER-97-552-s002.docx]

**Supplemental Table 2**: Microbial Indicator of Periodontitis and Risk of Mortality (n=5,037; NHANES 2009-2010, 2011-2012)

| **Microbial Indicator of Periodontitis (MIP)** | | | | | |
| --- | --- | --- | --- | --- | --- |
|  | **Per 1-Standard Deviation** | **Tertiles** | | | ^†^**Linear Trend** |
| Mean [min, max] MIP |  | Tertile 1  n = 1679  -4.61 [-11.89, -1.85] | Tertile 2  n = 1679  -0.78 [-1.85, 0.17] | Tertile 3  n = 1679  1.57 [0.17, 10.46] |  |
| **^*^CMD Mortality, HR (95% CI)** |  | n events = 23 | n events = 23 | n events = 35 |  |
| Model 1 | 1.92 (1.25, 2.96) | Ref. | 1.31 (0.55, 3.14) | 3.44 (1.46, 8.11) | <0.01 |
| Model 2 | 1.91 (1.17, 3.11) | Ref. | 1.14 (0.49, 2.63) | 3.10 (1.11, 8.63) | 0.03 |
| Model 3 | 1.95 (1.14, 3.33) | Ref. | 1.12 (0.52, 2.43) | 3.14 (1.07, 9.28) | 0.04 |
| Model 4 | 2.07 (1.21, 3.54) | Ref. | 1.23 (0.57, 2.67) | 3.87 (1.15, 13.01) | 0.03 |
| Model 5 | 2.10 (1.30, 3.38) | Ref. | 1.38 (0.65, 2.94) | 4.26 (1.32, 13.71) | 0.02 |
| **All-Cause Mortality, HR (95% CI)** |  | n events = 73 | n events = 90 | n events = 104 |  |
| Model 1 | 1.19 (0.94, 1.50) | Ref. | 1.08 (0.73, 1.58) | 1.49 (0.97, 2.28) | 0.07 |
| Model 2 | 1.15 (0.91, 1.47) | Ref. | 1.02 (0.67, 1.43) | 1.42 (0.84, 2.38) | 0.19 |
| Model 3 | 1.10 (0.85, 1.41) | Ref. | 0.86 (0.58, 1.26) | 1.24 (0.72, 2.14) | 0.44 |
| Model 4 | 1.04 (0.79, 1.37) | Ref. | 0.81 (0.54, 1.21) | 1.07 (0.58, 1.96) | 0.83 |
| Model 5 | 1.05 (0.79, 1.39) | Ref. | 0.83 (0.54, 1.27) | 1.09 (0.58, 2.03) | 0.79 |

* = Cardiometabolic Disease Mortality; † = linear trend p-value (p<0.05 = significant); HR = Hazard Ratio; CI = 95% confidence interval.

Hazard ratios and 95% confidence intervals were computed using survey-weighted multivariable proportional hazards regression.

Model 1: adjusts for survey cycle

Model 2: M1 + age + gender + race/ethnicity + education + income

Model 3: M2 + body mass index + Alternative Healthy Eating Index + physical activity + smoking history

Model 4: M3 + periodontal disease status (via CDC/AAP classification)

Model 5: M4 + HbA1c + systolic blood pressure + total cholesterol
