# Supplementary material for: Salivary microbiota and clinical periodontal measures predicting cardiometabolic disease mortality: A nationwide survey
Source: J Periodontol. 2025 Oct 10;97(3):552–68. doi: 10.1002/jper.11395 (PMC12934248; doi:10.1002/jper.11395)
Supplement: Supplementary file 10 — Supporting Information [file JPER-97-552-s011.docx]

**Supplemental Table 3**: Periodontal Disease Measures and Risk of Mortality (n=5,037; NHANES 2009-2010, 2011-2012)

| **Interproximal Periodontal Probing Depth (I-PPD)** | | | | | | | | |
| --- | --- | --- | --- | --- | --- | --- | --- | --- |
|  | **Per 1-Standard Deviation** | **Tertiles** | | | | | | ^†^**Linear Trend** |
| Mean [min, max] I-PPD, mm |  | Tertile 1  n = 1679  1.20 [0.11, 1.46] | | Tertile 2  n = 1675  1.70 [1.46, 1.97] | | Tertile 3  n = 1683  2.65 [1.97, 6.08] | |  |
| **^*^CMD Mortality, HR (95% CI)** |  | n events = 14 | | n events = 28 | | n events = 39 | |  |
| Model 1 | 1.44 (1.22, 1.71) | Ref. | | 2.11 (0.70, 6.34) | | 3.20 (1.40, 7.31) | | 0.01 |
| Model 2 | 1.06 (0.85, 1.32) | Ref. | | 1.51 (0.54, 4.27) | | 1.49 (0.69, 3.21) | | 0.31 |
| Model 3 | 1.08 (0.87, 1.35) | Ref. | | 1.65 (0.56, 4.84) | | 1.57 (0.73, 3.36) | | 0.25 |
| Model 4 | 1.24 (0.82, 1.87) | Ref. | | 1.69 (0.58, 4.92) | | 1.64 (0.74, 3.65) | | 0.23 |
| Model 5 | 1.23 (0.80, 1.89) | Ref. | | 1.65 (0.58, 4.67) | | 1.60 (0.70, 3.64) | | 0.27 |
| **All-Cause Mortality, HR (95% CI)** |  | n events = 54 | | n events = 86 | | n events = 127 | |  |
| Model 1 | 1.37 (1.21, 1.55) | Ref. | | 1.59 (0.88, 2.87) | | 2.60 (1.63, 4.14) | | <0.01 |
| Model 2 | 1.11 (0.94, 1.30) | Ref. | | 1.27 (0.71, 2.25) | | 1.59 (0.97, 2.60) | | 0.07 |
| Model 3 | 1.05 (0.89, 1.25) | Ref. | | 1.27 (0.71, 2.28) | | 1.41 (0.88, 2.26) | | 0.16 |
| Model 4 | 0.90 (0.72, 1.11) | Ref. | | 1.27 (0.71, 2.28) | | 1.41 (0.88, 2.26) | | 0.16 |
| Model 5 | 0.89 (0.72, 1.10) | Ref. | | 1.27 (0.72, 2.26) | | 1.37 (0.84, 2.22) | | 0.21 |
| **Interproximal Clinical Attachment Loss (I-CAL)** | | | | | | | | |
|  | **Per 1-Standard Deviation** | **Tertiles** | | | | | | ^†^**Linear Trend** |
| Mean [min, max] I-CAL, mm |  | Tertile 1  n = 1678  0.88 [0.01, 1.17] | | Tertile 2  n = 1679  1.46 [1.17, 1.85] | | Tertile 3  n = 1680  3.07 [1.85, 12.33] | |  |
| **^*^CMD Mortality, HR (95% CI)** |  | n events = 10 | | n events = 23 | | n events = 48 | |  |
| Model 1 | 1.47 (1.31, 1.64) | Ref. | | 2.24 (1.16, 4.34) | | 3.97 (2.28, 6.93) | | <0.01 |
| Model 2 | 0.95 (0.80, 1.14) | Ref. | | 1.20 (0.63, 2.30) | | 1.15 (0.57, 2.33) | | 0.69 |
| Model 3 | 0.95 (0.80, 1.13) | Ref. | | 1.21 (0.64, 2.27) | | 1.14 (0.63, 2.07) | | 0.67 |
| Model 4 | 1.01 (0.79, 1.30) | Ref. | | 1.22 (0.63, 2.36) | | 1.16 (0.60, 2.25) | | 0.65 |
| Model 5 | 1.00 (0.78, 1.30) | Ref. | | 1.18 (0.59, 2.33) | | 1.06 (0.53, 2.14) | | 0.87 |
| **All-Cause Mortality, HR (95% CI)** |  | n events = 45 | | n events = 67 | | n events = 155 | |  |
| Model 1 | 1.60 (1.45, 1.77) | Ref. | | 1.89 (1.20, 2.97) | | 5.01 (3.49, 7.19) | | <0.01 |
| Model 2 | 1.27 (1.10, 1.48) | Ref. | | 1.32 (0.83, 2.10) | | 2.51 (1.50, 4.21) | | <0.01 |
| Model 3 | 1.21 (1.05, 1.41) | Ref. | | 1.27 (0.80, 2.04) | | 2.19 (1.32, 3.64) | | <0.01 |
| Model 4 | 1.15 (0.95, 1.39) | Ref. | | 1.27 (0.80, 2.04) | | 2.19 (1.32, 3.64) | | <0.01 |
| Model 5 | 1.13 (0.94, 1.36) | Ref. | | 1.26 (0.79, 2.02) | | 2.10 (1.25, 3.52) | | <0.01 |
| **Periodontal Disease Status (via CDC/AAP classification)** | | | | | | | | |
|  | **CDC/AAP Categories** | | | | | | ^†^**Linear Trend** | |
|  | Healthy/Mild  n = 3636 | | Moderate  n = 2551 | | Severe  n = 879 | |  | |
| **^*^CMD Mortality, HR (95% CI)** | n events = 69 | | n events = 122 | | n events = 43 | |  | |
| Model 1 | Ref. | | 1.54 (0.71, 3.37) | | 2.75 (1.21, 6.24) | | 0.02 | |
| Model 2 | Ref. | | 0.61 (0.25, 1.46) | | 0.74 (0.30, 1.83) | | 0.51 | |
| Model 3 | Ref. | | 0.56 (0.22, 1.45) | | 0.75 (0.29, 1.95) | | 0.56 | |
| Model 4 | Ref. | | 0.56 (0.21, 1.48) | | 0.75 (0.32, 1.79) | | 0.52 | |
| Model 5 | Ref. | | 0.51 (0.19, 1.36) | | 0.72 (0.31, 1.69) | | 0.46 | |
| **All-Cause Mortality, HR (95% CI)** | n events = 201 | | n events = 371 | | n events = 128 | |  | |
| Model 1 | Ref. | | 2.68 (1.74, 4.14) | | 4.55 (2.74, 7.54) | | <0.01 | |
| Model 2 | Ref. | | 1.58 (0.93, 2.69) | | 2.22 (1.20, 4.08) | | 0.01 | |
| Model 3 | Ref. | | 1.40 (0.82, 2.38) | | 1.94 (1.02, 3.68) | | 0.04 | |
| Model 4 | Ref. | | 1.40 (0.82, 2.38) | | 1.94 (1.02, 3.68) | | 0.04 | |
| Model 5 | Ref. | | 1.35 (0.79, 2.30) | | 1.84 (0.95, 3.57) | | 0.07 | |

* = Cardiometabolic Disease Mortality; † = p-value for linear trend across tertiles; HR = Hazard Ratio; CI = 95% confidence interval. CDC/AAP = Centers for Disease Control/American Academy of Periodontology; CAL = Clinical Attachment Loss; PPD = Periodontal Probing Depth.

Hazard ratios and 95% confidence intervals were computed using survey-weighted multivariable proportional hazards regression.

Model 1: adjusts for survey cycle

Model 2: M1 + age + gender + race/ethnicity + education + income

Model 3: M2 + body mass index + Alternative Healthy Eating Index + physical activity + smoking history

Model 4: M3 + Shannon Diversity Index

Model 5: M4 + baseline HbA1c + baseline systolic blood pressure + baseline total cholesterol
