# Supplementary material for: Salivary microbiota and clinical periodontal measures predicting cardiometabolic disease mortality: A nationwide survey
Source: J Periodontol. 2025 Oct 10;97(3):552–68. doi: 10.1002/jper.11395 (PMC12934248; doi:10.1002/jper.11395)
Supplement: Supplementary file 11 — Supporting Information [file JPER-97-552-s006.docx]

**Supplemental Table 4**: Microbial Indicator of Periodontitis and Prevalence of Moderate/Severe Periodontal Disease (n=5,037; NHANES 2009-2010, 2011-2012)

| **Microbial Indicator of Periodontitis (MIP)** | | | | | |
| --- | --- | --- | --- | --- | --- |
|  | **Per 1-Standard Deviation** | **Tertiles** | | | ^†^**Linear Trend** |
| Mean [min, max] MIP |  | Tertile 1  n = 1679  -4.61 [-11.89, -1.85] | Tertile 2  n = 1679  -0.78 [-1.85, 0.17] | Tertile 3  n = 1679  1.57 [0.17, 10.46] |  |
| ***Moderate/Severe Periodontal Disease, PR (95% CI)** |  | n with disease = 463 | n with disease = 736 | n with disease = 1057 |  |
| Model 1 | 1.44 (1.34, 1.54) | Ref. | 1.74 (1.48, 2.04) | 2.69 (2.21, 3.28) | *p* < 0.01 |
| Model 2 | 1.34 (1.26, 1.42) | Ref. | 1.55 (1.33, 1.80) | 2.27 (1.92, 2.68) | *p* < 0.01 |
| Model 3 | 1.30 (1.22, 1.38) | Ref. | 1.48 (1.26, 1.73) | 2.10 (1.76, 2.51) | *p* < 0.01 |
| Model 4 | 1.29 (1.22, 1.39) | Ref. | 1.48 (1.26, 1.75) | 2.10 (1.74, 2.53) | *p* < 0.01 |

* = Defined as moderate/severe periodontal disease via CDC/AAP classification (healthy/mild disease set as reference); † = linear trend p-value (p<0.05 = significant); PR = Prevalence Ratio; CI = 95% confidence interval.

Prevalence ratios and 95% confidence intervals were computed using survey-weighted multivariable robust variance Poisson regression models.

Model 1: adjusts for survey cycle

Model 2: M1 + age + gender + race/ethnicity + education + income

Model 3: M2 + body mass index + Alternative Healthy Eating Index + physical activity + smoking history

Model 4: M3 + HbA1c + systolic blood pressure + total cholesterol
