# Supplementary material for: Salivary microbiota and clinical periodontal measures predicting cardiometabolic disease mortality: A nationwide survey
Source: J Periodontol. 2025 Oct 10;97(3):552–68. doi: 10.1002/jper.11395 (PMC12934248; doi:10.1002/jper.11395)
Supplement: Supplementary file 12 — Supporting Information [file JPER-97-552-s012.docx]

**Supplemental Table 5**: Oral Microbial Indices and Mortality Risk Stratified by Periodontal Disease Status (n=5,037; NHANES 2009-2010, 2011-2012)

|  | **CMD Mortality, HR (95% CI)** | | **All-Cause Mortality, HR (95% CI)** | |
| --- | --- | --- | --- | --- |
| Periodontal Disease | n events / n total | Per 1-SD of MIP | n events / n total | Per 1-SD of MIP |
| Healthy/Mild | 27 / 2781 | 2.93 (1.60, 5.39) | 88 / 2781 | 1.38 (0.91, 2.08) |
| Moderate | 37 / 1626 | 1.32 (0.78, 2.22) | 116 / 1626 | 0.88 (0.57, 1.36) |
| Severe | 17 / 630 | 1.23 (0.62, 2.43) | 63 / 630 | 0.81 (0.56, 0.90) |
| MIP x Periodontal  Disease Interaction |  | *p* = 0.15 |  | *p* = 0.16 |
|  | **CMD Mortality, HR (95% CI)** | | **All-Cause Mortality, HR (95% CI)** | |
| Periodontal Disease | n events / n total | Per 1-SD of Shannon  Index | n events / n total | Per 1-SD of Shannon Index |
| Healthy/Mild | 27 / 2781 | 0.75 (0.44, 1.26) | 88 / 2781 | 0.73 (0.54, 0.99) |
| Moderate | 37 / 1626 | 0.77 (0.56, 1.06) | 116 / 1626 | 0.85 (0.66, 1.09) |
| Severe | 17 / 630 | 1.53 (0.96, 2.42) | 63 / 630 | 0.61 (0.45, 0.82) |
| Shannon Index x Periodontal  Disease Interaction |  | *p* = 0.14 |  | *p* = 0.19 |

CMD = Cardiometabolic Disease Mortality; HR = Hazard Ratio; CI = 95% confidence interval; SD = standard deviation; interaction *p*-values<0.05 are statistically significant.

Hazard ratios and 95% confidence intervals were computed using survey-weighted multivariable proportional hazards regression.

MIP models stratified by periodontal disease adjust for survey cycle, age, gender, race/ethnicity, education, income, body mass index, Alternative Healthy Eating Index, physical activity, smoking history, HbA1c, systolic blood pressure, and total cholesterol.

Shannon diversity index models stratified by periodontal disease adjust for survey cycle, age, gender, race/ethnicity, education, income, body mass index, Alternative Healthy Eating Index, physical activity, smoking history, HbA1c, systolic blood pressure, and total cholesterol.

MIP x periodontal disease interaction models adjust for survey cycle, age, gender, race/ethnicity, education, income, body mass index, Alternative Healthy Eating Index, physical activity, smoking history, HbA1c, systolic blood pressure, and total cholesterol.

Shannon diversity index x periodontal disease interaction models adjust for survey cycle, age, gender, race/ethnicity, education, income, body mass index, Alternative Healthy Eating Index, physical activity, smoking history, HbA1c, systolic blood pressure, and total cholesterol.
