# Supplementary material for: Salivary microbiota and clinical periodontal measures predicting cardiometabolic disease mortality: A nationwide survey
Source: J Periodontol. 2025 Oct 10;97(3):552–68. doi: 10.1002/jper.11395 (PMC12934248; doi:10.1002/jper.11395)
Supplement: Supplementary file 13 — Supporting Information [file JPER-97-552-s015.docx]

**Supplemental Table 6**: β-Diversity PCoA Axis 1 and Risk of Mortality (n=5,037; NHANES 2009-2010, 2011-2012)

| **Weighted UniFrac PCoA Axis 1** | | | | | |
| --- | --- | --- | --- | --- | --- |
|  | **Per 1-Standard Deviation** | **Tertiles** | | | ^†^**Linear Trend** |
| Mean [min, max] Weighted UniFrac |  | Tertile 1  n = 1679  -0.13 [-0.42, -0.06] | Tertile 2  n = 1679  0.00 [-0.06, 0.05] | Tertile 3  n = 1679  0.13 [0.05, 0.52] |  |
| **^*^CMD Mortality, HR (95% CI)** |  | n events = 22 | n events = 31 | n events = 28 |  |
| Model 1 | 1.15 (0.88, 1.50) | Ref. | 1.46 (0.62, 3.43) | 1.64 (0.68, 3.98) | 0.28 |
| Model 2 | 1.17 (0.91, 1.51) | Ref. | 1.57 (0.65, 3.81) | 1.79 (0.74, 4.28) | 0.19 |
| Model 3 | 1.17 (0.90, 1.52) | Ref. | 1.46 (0.55, 3.91) | 1.81 (0.71, 4.63) | 0.22 |
| Model 4 | 1.16 (0.91, 1.50) | Ref. | 1.44 (0.53, 3.87) | 1.78 (0.71, 4.45) | 0.22 |
| Model 5 | 1.11 (0.86, 1.44) | Ref. | 1.41 (0.51, 3.88) | 1.73 (0.67, 4.43) | 0.26 |
| **All-Cause Mortality, HR (95% CI)** |  | n events = 73 | n events = 86 | n events = 108 |  |
| Model 1 | 1.31 (1.08, 1.58) | Ref. | 0.97 (0.61, 1.55) | 1.51 (0.88, 2.59) | 0.14 |
| Model 2 | 1.30 (1.09, 1.56) | Ref. | 1.00 (0.61, 1.64) | 1.54 (0.92, 2.59) | 0.10 |
| Model 3 | 1.27 (1.06, 1.52) | Ref. | 0.96 (0.59, 1.58) | 1.46 (0.86, 2.49) | 0.16 |
| Model 4 | 1.31 (1.09, 1.57) | Ref. | 1.01 (0.61, 1.65) | 1.59 (0.93, 2.72) | 0.09 |
| Model 5 | 1.29 (1.07, 1.56) | Ref. | 1.00 (0.62, 1.63) | 1.58 (0.93, 2.69) | 0.09 |
| **Unweighted UniFrac PCoA Axis 1** | | | | | |
|  | **Per 1-Standard Deviation** | **Tertiles** | | | ^†^**Linear Trend** |
| Mean [min, max] Unweighted UniFrac |  | Tertile 1  n = 1679  -0.15 [-0.31, -0.07] | Tertile 2  n = 1679  0.00 [-0.07, 0.70] | Tertile 3  n = 1679  0.15 [0.07, 0.33] |  |
| **^*^CMD Mortality, HR (95% CI)** |  | n events = 28 | n events = 27 | n events = 26 |  |
| Model 1 | 0.81 (0.59, 1.11) | Ref. | 0.43 (0.18, 1.02) | 0.55 (0.24, 1.24) | 0.15 |
| Model 2 | 0.86 (0.57, 1.31) | Ref. | 0.46 (0.15, 1.39) | 0.65 (0.23, 1.80) | 0.40 |
| Model 3 | 0.85 (0.53, 1.36) | Ref. | 0.41 (0.13, 1.27) | 0.63 (0.21, 1.91) | 0.42 |
| Model 4 | 0.78 (0.46, 1.31) | Ref. | 0.34 (0.10, 1.20) | 0.49 (0.14, 1.76) | 0.28 |
| Model 5 | 0.74 (0.45, 1.23) | Ref. | 0.33 (0.10, 1.11) | 0.44 (0.12, 1.58) | 0.21 |
| **All-Cause Mortality, HR (95% CI)** |  | n events = 96 | n events = 76 | n events = 95 |  |
| Model 1 | 0.96 (0.80, 1.16) | Ref. | 0.61 (0.41, 0.90) | 0.85 (0.57, 1.26) | 0.41 |
| Model 2 | 0.98 (0.81, 1.19) | Ref. | 0.62 (0.40, 0.97) | 0.90 (0.60, 1.35) | 0.61 |
| Model 3 | 1.03 (0.83, 1.27) | Ref. | 0.62 (0.39, 1.00) | 1.00 (0.65, 1.55) | 0.99 |
| Model 4 | 1.14 (0.87, 1.49) | Ref. | 0.73 (0.44, 1.23) | 1.23 (0.69, 2.18) | 0.48 |
| Model 5 | 1.13 (0.86, 1.48) | Ref. | 0.72 (0.43, 1.22) | 1.20 (0.64, 2.22) | 0.57 |
| **Bray-Curtis Dissimilarity PCoA Axis 1** | | | | | |
|  | **Per 1-Standard Deviation** | **Tertiles** | | | ^†^**Linear Trend** |
| Mean [min, max] Bray-Curtis |  | Tertile 1  n = 1679  -0.19 [-0.43, -0.08] | Tertile 2  n = 1679  0.00 [-0.08, 0.08] | Tertile 3  n = 1679  0.19 [0.08, 0.44] |  |
| **^*^CMD Mortality, HR (95% CI)** |  | n events = 24 | n events = 31 | n events = 26 |  |
| Model 1 | 1.17 (0.83, 1.66) | Ref. | 1.09 (0.54, 2.20) | 1.38 (0.61, 3.17) | 0.44 |
| Model 2 | 1.05 (0.74, 1.50) | Ref. | 0.97 (0.46, 2.05) | 1.08 (0.47, 2.51) | 0.85 |
| Model 3 | 1.05 (0.71, 1.54) | Ref. | 0.93 (0.45, 1.91) | 1.05 (0.44, 2.51) | 0.91 |
| Model 4 | 1.05 (0.72, 1.55) | Ref. | 0.95 (0.45, 1.99) | 1.07 (0.45, 2.55) | 0.88 |
| Model 5 | 1.04 (0.72, 1.51) | Ref. | 0.95 (0.46, 1.97) | 1.06 (0.45, 2.50) | 0.90 |
| **All-Cause Mortality, HR (95% CI)** |  | n events = 68 | n events = 98 | n events = 101 |  |
| Model 1 | 1.22 (1.02, 1.46) | Ref. | 1.34 (0.91, 1.97) | 1.56 (1.01, 2.41) | 0.05 |
| Model 2 | 1.10 (0.92, 1.33) | Ref. | 1.20 (0.80, 1.79) | 1.22 (0.78, 1.92) | 0.39 |
| Model 3 | 1.04 (0.87, 1.24) | Ref. | 1.10 (0.73, 1.68) | 1.06 (0.68, 1.65) | 0.80 |
| Model 4 | 1.05 (0.87, 1.26) | Ref. | 1.10 (0.73, 1.66) | 1.08 (0.69, 1.68) | 0.75 |
| Model 5 | 1.04 (0.87, 1.25) | Ref. | 1.10 (0.72, 1.66) | 1.07 (0.68, 1.66) | 0.78 |

* = Cardiometabolic Disease Mortality; † = linear trend p-value (p<0.05 = significant); HR = Hazard Ratio; CI = 95% confidence interval; PCoA = Principal Coordinates Analysis.

Hazard ratios and 95% confidence intervals were computed using survey-weighted multivariable proportional hazards regression.

Model 1: adjusts for survey cycle

Model 2: M1 + age + gender + race/ethnicity + education + income

Model 3: M2 + body mass index + Alternative Healthy Eating Index + physical activity + smoking history

Model 4: M3 + periodontal disease status (via CDC/AAP classification)

Model 5: M4 + HbA1c + systolic blood pressure + total cholesterol
