# Supplementary material for: Salivary microbiota and clinical periodontal measures predicting cardiometabolic disease mortality: A nationwide survey
Source: J Periodontol. 2025 Oct 10;97(3):552–68. doi: 10.1002/jper.11395 (PMC12934248; doi:10.1002/jper.11395)
Supplement: Supplementary file 14 — Supporting Information [file JPER-97-552-s005.docx]

**Supplemental Table 7**: Salivary α-Diversity and Risk of Mortality (n=5,037; NHANES 2009-2010, 2011-2012)

| **Observed Amplicon Sequencing Variants (ASVs)** | | | | | |
| --- | --- | --- | --- | --- | --- |
|  | **Per 1-Standard Deviation** | **Tertiles** | | | ^†^**Linear Trend** |
| Mean [min, max] Observed ASVs |  | Tertile 1  n = 1679  88.35 [13, 111] | Tertile 2  n = 1678  128.52 [111, 146] | Tertile 3  n = 1680  180.93 [147, 348] |  |
| **^*^CMD Mortality, HR (95% CI)** |  | n events = 30 | n events = 26 | n events = 25 |  |
| Model 1 | 0.98 (0.70, 1.36) | Ref. | 0.72 (0.36, 1.45) | 0.98 (0.46, 2.10) | 0.95 |
| Model 2 | 0.93 (0.64, 1.35) | Ref. | 0.74 (0.38, 1.44) | 0.86 (0.34, 2.18) | 0.75 |
| Model 3 | 0.94 (0.62, 1.42) | Ref. | 0.73 (0.36, 1.50) | 0.88 (0.33, 2.31) | 0.79 |
| Model 4 | 0.97 (0.65, 1.43) | Ref. | 0.73 (0.36, 1.48) | 0.96 (0.37, 2.47) | 0.93 |
| Model 5 | 0.99 (0.67, 1.45) | Ref. | 0.74 (0.37, 1.46) | 0.97 (0.39, 2.44) | 0.95 |
| **All-Cause Mortality, HR (95% CI)** |  | n events = 101 | n events = 85 | n events = 81 |  |
| Model 1 | 0.88 (0.74, 1.05) | Ref. | 0.78 (0.51, 1.20) | 0.81 (0.56, 1.16) | 0.24 |
| Model 2 | 0.87 (0.74, 1.04) | Ref. | 0.81 (0.53, 1.24) | 0.78 (0.55, 1.12) | 0.18 |
| Model 3 | 0.87 (0.73, 1.04) | Ref. | 0.83 (0.54, 1.27) | 0.78 (0.54, 1.12) | 0.17 |
| Model 4 | 0.80 (0.65, 0.98) | Ref. | 0.78 (0.50, 1.22) | 0.64 (0.42, 0.98) | 0.04 |
| Model 5 | 0.80 (0.65, 0.99) | Ref. | 0.78 (0.50, 1.22) | 0.65 (0.41, 1.01) | 0.06 |
| **Shannon Diversity Index** | | | | | |
|  | **Per 1-Standard Deviation** | **Tertiles** | | | ^†^**Linear Trend** |
| Mean [min, max] Shannon Index |  | Tertile 1  n = 1679  3.92 [0.5, 4.41] | Tertile 2  n = 1679  4.68 [4.41, 4.93] | Tertile 3  n = 1679  5.33 [4.93, 6.6] |  |
| **^*^CMD Mortality, HR (95% CI)** |  | n events = 29 | n events = 27 | n events = 25 |  |
| Model 1 | 0.87 (0.66, 1.14) | Ref. | 0.90 (0.38, 2.13) | 1.04 (0.48, 2.27) | 0.92 |
| Model 2 | 0.81 (0.60, 1.10) | Ref. | 0.90 (0.38, 2.16) | 0.89 (0.37, 2.13) | 0.80 |
| Model 3 | 0.81 (0.59, 1.12) | Ref. | 0.96 (0.40, 2.27) | 0.93 (0.36, 2.38) | 0.87 |
| Model 4 | 0.82 (0.60, 1.11) | Ref. | 0.99 (0.41, 2.39) | 1.00 (0.40, 2.50) | 1.00 |
| Model 5 | 0.84 (0.63, 1.14) | Ref. | 1.03 (0.42, 2.51) | 1.00 (0.39, 2.56) | 0.99 |
| **All-Cause Mortality, HR (95% CI)** |  | n events = 103 | n events = 85 | n events = 79 |  |
| Model 1 | 0.83 (0.69, 0.99) | Ref. | 0.68 (0.41, 1.12) | 0.76 (0.47, 1.20) | 0.24 |
| Model 2 | 0.79 (0.66, 0.96) | Ref. | 0.68 (0.42, 1.12) | 0.69 (0.43, 1.13) | 0.14 |
| Model 3 | 0.80 (0.66, 0.97) | Ref. | 0.73 (0.45, 1.20) | 0.71 (0.43, 1.16) | 0.17 |
| Model 4 | 0.76 (0.63, 0.92) | Ref. | 0.69 (0.40, 1.17) | 0.60 (0.35, 1.03) | 0.06 |
| Model 5 | 0.77 (0.63, 0.94) | Ref. | 0.68 (0.40, 1.18) | 0.60 (0.35, 1.04) | 0.07 |
| **Inverse Simpson Diversity Index** | | | | | |
|  | **Per 1-Standard Deviation** | **Tertiles** | | | ^†^**Linear Trend** |
| Mean [min, max] Inv. Simp. Index |  | Tertile 1  n = 1679  0.84 [0.10, 0.90] | Tertile 2  n = 1679  0.92 [0.90, 0.93] | Tertile 3  n = 1679  0.95 [0.93, 0.98] |  |
| **^*^CMD Mortality, HR (95% CI)** |  | n events = 35 | n events = 25 | n events = 21 |  |
| Model 1 | 0.93 (0.76, 1.13) | Ref. | 0.60 (0.27, 1.34) | 0.65 (0.27, 1.54) | 0.32 |
| Model 2 | 0.88 (0.71, 1.09) | Ref. | 0.57 (0.25, 1.32) | 0.56 (0.22, 1.45) | 0.23 |
| Model 3 | 0.86 (0.71, 1.05) | Ref. | 0.54 (0.24, 1.23) | 0.57 (0.21, 1.52) | 0.26 |
| Model 4 | 0.86 (0.71, 1.05) | Ref. | 0.54 (0.24, 1.24) | 0.58 (0.23, 1.49) | 0.26 |
| Model 5 | 0.88 (0.73, 1.05) | Ref. | 0.60 (0.27, 1.37) | 0.58 (0.23, 1.48) | 0.26 |
| **All-Cause Mortality, HR (95% CI)** |  | n events = 100 | n events = 89 | n events = 78 |  |
| Model 1 | 0.92 (0.80, 1.06) | Ref. | 0.76 (0.49, 1.19) | 0.72 (0.44, 1.18) | 0.19 |
| Model 2 | 0.88 (0.76, 1.02) | Ref. | 0.72 (0.45, 1.16) | 0.66 (0.39, 1.10) | 0.11 |
| Model 3 | 0.88 (0.77, 1.01) | Ref. | 0.71 (0.44, 1.13) | 0.67 (0.40, 1.13) | 0.14 |
| Model 4 | 0.87 (0.76, 0.99) | Ref. | 0.70 (0.44, 1.13) | 0.61 (0.36, 1.02) | 0.06 |
| Model 5 | 0.88 (0.76, 1.00) | Ref. | 0.72 (0.44, 1.16) | 0.61 (0.36, 1.03) | 0.06 |
| **Faith's Phylogenetic Diversity Index** | | | | | |
|  | **Per 1-Standard Deviation** | **Tertiles** | | | ^†^**Linear Trend** |
| Mean [min, max] Faith's Index |  | Tertile 1  n = 1679  11.23 [2.82, 3.12] | Tertile 2  n = 1679  14.50 [13.12, 15.98] | Tertile 3  n = 1679  18.46 [15.98, 31.33] |  |
| **^*^CMD Mortality, HR (95% CI)** |  | n events = 28 | n events = 28 | n events = 25 |  |
| Model 1 | 1.05 (0.77, 1.43) | Ref. | 0.94 (0.51, 1.75) | 1.24 (0.58, 2.65) | 0.58 |
| Model 2 | 0.98 (0.69, 1.40) | Ref. | 0.89 (0.47, 1.66) | 1.04 (0.42, 2.56) | 0.93 |
| Model 3 | 1.00 (0.67, 1.48) | Ref. | 0.82 (0.40, 1.69) | 1.04 (0.40, 2.70) | 0.94 |
| Model 4 | 1.04 (0.70, 1.53) | Ref. | 0.84 (0.42, 1.71) | 1.17 (0.47, 2.92) | 0.74 |
| Model 5 | 1.07 (0.73, 1.57) | Ref. | 0.91 (0.47, 1.73) | 1.25 (0.51, 3.06) | 0.63 |
| **All-Cause Mortality, HR (95% CI)** |  | n events = 93 | n events = 91 | n events = 83 |  |
| Model 1 | 0.92 (0.78, 1.08) | Ref. | 0.96 (0.67, 1.38) | 0.95 (0.66, 1.38) | 0.79 |
| Model 2 | 0.90 (0.77, 1.05) | Ref. | 0.92 (0.62, 1.37) | 0.88 (0.59, 1.31) | 0.53 |
| Model 3 | 0.88 (0.74, 1.03) | Ref. | 0.86 (0.57, 1.30) | 0.83 (0.55, 1.26) | 0.38 |
| Model 4 | 0.80 (0.66, 0.97) | Ref. | 0.80 (0.52, 1.25) | 0.68 (0.41, 1.12) | 0.13 |
| Model 5 | 0.81 (0.66, 0.99) | Ref. | 0.81 (0.52, 1.26) | 0.69 (0.41, 1.17) | 0.17 |

* = Cardiometabolic Disease Mortality; †= p-value for linear trend across tertiles; HR = Hazard Ratio; CI = 95% confidence interval.

Hazard ratios and 95% confidence intervals were computed using survey-weighted multivariable proportional hazards regression.

Model 1: adjusts for survey cycle

Model 2: M1 + age + gender + race/ethnicity + education + income

Model 3: M2 + body mass index + Alternative Healthy Eating Index + physical activity + smoking history

Model 4: M3 + periodontal disease status (via CDC/AAP classification)

Model 5: M4 + HbA1c + systolic blood pressure + total cholesterol
