# Supplementary material for: Salivary microbiota and clinical periodontal measures predicting cardiometabolic disease mortality: A nationwide survey
Source: J Periodontol. 2025 Oct 10;97(3):552–68. doi: 10.1002/jper.11395 (PMC12934248; doi:10.1002/jper.11395)
Supplement: Supplementary file 15 — Supporting Information [file JPER-97-552-s004.docx]

**Supplemental Table 8**: Differential Abundance Analysis of Microbial Taxa by Periodontal Disease Status (n=5,037; NHANES 2009-2010, 2011-2012)

| **Taxonomic Name** | **Log-fold-change (LFC)** | **Standard Error of LFC** | **Raw p-value** | **FDR q-value** |
| --- | --- | --- | --- | --- |
| **Leptotrichiaceae (family)** | **1.146350778** | **0.067933994** | **4.74792E-57** | **5.74498E-55** |
| **Filifactor** | **0.934109269** | **0.062252484** | **4.02716E-49** | **2.43643E-47** |
| **Treponema_2** | **0.919009966** | **0.061731085** | **6.12041E-49** | **2.46857E-47** |
| **Prevotellaceae (family)** | **0.73303505** | **0.064469618** | **4.61197E-29** | **1.39512E-27** |
| **Incertae_Sedis** | **0.691720386** | **0.06256537** | **1.59179E-27** | **3.85213E-26** |
| **Defluviitaleaceae_UCG-011** | **0.637044086** | **0.061580088** | **1.87446E-24** | **3.24014E-23** |
| **Fretibacterium** | **0.62130882** | **0.059972193** | **9.25681E-25** | **1.86679E-23** |
| **Mycoplasma** | **0.585311633** | **0.061641622** | **4.05759E-21** | **6.1371E-20** |
| **Bacteroidetes (phylum)** | **0.526106429** | **0.063213812** | **1.83658E-16** | **2.22226E-15** |
| **Peptococcus** | **0.468012846** | **0.059446773** | **4.62581E-15** | **5.08839E-14** |
| **Tannerella** | **0.462653095** | **0.055041192** | **5.7768E-17** | **7.76659E-16** |
| **Family_XIII (family)** | **0.452548268** | **0.059547635** | **4.32787E-14** | **4.36393E-13** |
| **Family_XI (family)** | **0.437137604** | **0.06348526** | **1.67082E-11** | **1.3478E-10** |
| **Desulfobulbus** | **0.428778196** | **0.062568506** | **1.04156E-11** | **9.00207E-11** |
| **Clostridiales_vadinBB60_group (family)** | **0.410645639** | **0.060857248** | **2.05464E-11** | **1.55382E-10** |
| **Porphyromonas** | **0.407521125** | **0.058336482** | **3.21181E-12** | **2.98946E-11** |
| **Peptostreptococcus** | **0.392221145** | **0.059537896** | **4.98805E-11** | **3.55032E-10** |
| **Bacteroidales (order)** | **0.375059013** | **0.060685355** | **7.48605E-10** | **4.76743E-09** |
| **NB1-n (order)** | **0.371151452** | **0.063010383** | **6.35726E-09** | **3.66299E-08** |
| **Peptoclostridium** | **0.359345882** | **0.058711331** | **1.06821E-09** | **6.46265E-09** |
| **Parvimonas** | **0.356219228** | **0.056406432** | **2.94182E-10** | **1.97756E-09** |
| **Selenomonas** | **0.327679404** | **0.05756622** | **1.35285E-08** | **7.44066E-08** |
| **[Eubacterium]_brachy_group** | **0.28595695** | **0.058582898** | **1.09549E-06** | **5.52308E-06** |
| **WCHB1-69 (family)** | **0.280511117** | **0.062047144** | **6.56607E-06** | **2.94257E-05** |
| **Family_XIII_UCG-001** | **0.25639676** | **0.0586536** | **1.2909E-05** | **5.38619E-05** |
| **[Eubacterium]_nodatum_group** | **0.240052355** | **0.055964292** | **1.82917E-05** | **7.13966E-05** |
| **Aggregatibacter** | **0.238887844** | **0.06070898** | **8.48218E-05** | **0.000293241** |
| **Desulfovibrio** | **0.233582708** | **0.063464278** | **0.000255205** | **0.000812627** |
| **Dialister** | **0.221587121** | **0.054703831** | **5.19126E-05** | **0.000190346** |
| **Fusobacteriales (order)** | **0.206769392** | **0.064857193** | **0.001504589** | **0.003792818** |
| **Prevotella_2** | **0.199891618** | **0.053861459** | **0.000208797** | **0.000682824** |
| **Actinomycetaceae (family)** | **0.193035068** | **0.057452778** | **0.000788019** | **0.002325617** |
| **Streptobacillus** | **0.190588691** | **0.065919213** | **0.00391709** | **0.009293488** |
| **Acholeplasma** | **0.182981637** | **0.065457107** | **0.005379808** | **0.011420294** |
| **Selenomonas_4** | **0.179554971** | **0.060098528** | **0.002846774** | **0.006889194** |
| **Eggerthia** | **0.17885193** | **0.06242053** | **0.004225145** | **0.009831586** |
| **Catonella** | **0.170890797** | **0.053456955** | **0.001399738** | **0.003666116** |
| **Fusobacterium** | **0.166283307** | **0.048708144** | **0.000645576** | **0.001981937** |
| **Olsenella** | **0.154565835** | **0.060609207** | **0.010851577** | **0.020516263** |
| **Mitochondria (family)** | **0.149054879** | **0.06718259** | **0.026670889** | **0.047458493** |
| **Mogibacterium** | **0.142599355** | **0.053165888** | **0.007339937** | **0.015221382** |
| **Porphyromonadaceae (family)** | **0.137712507** | **0.062640285** | **0.027972193** | **0.047852543** |
| Phocaeicola | 0.131503811 | 0.06160715 | 0.032930328 | 0.054583146 |
| Pasteurellaceae (family) | 0.126661338 | 0.06605242 | 0.055451327 | 0.085018164 |
| Johnsonella | 0.106659941 | 0.061072477 | 0.080888257 | 0.119359501 |
| Anaeroglobus | 0.101655218 | 0.060923735 | 0.09529688 | 0.137272887 |
| Campylobacter | 0.096654424 | 0.048041881 | 0.044286849 | 0.070509326 |
| Prevotella | 0.090919518 | 0.049718596 | 0.067506807 | 0.102104046 |
| Bulleidia | 0.089087828 | 0.061725844 | 0.149082004 | 0.202684522 |
| Pseudoramibacter | 0.083279845 | 0.062671723 | 0.184108957 | 0.236991317 |
| Selenomonas_3 | 0.076949385 | 0.05483338 | 0.160587416 | 0.213528322 |
| Slackia | 0.07520865 | 0.062083833 | 0.226101638 | 0.276346447 |
| Alloprevotella | 0.074105972 | 0.057096181 | 0.194377323 | 0.247575327 |
| Shuttleworthia | 0.071719354 | 0.060246278 | 0.234010226 | 0.283152374 |
| Erysipelotrichaceae_UCG-006 | 0.055432038 | 0.060698351 | 0.36125843 | 0.424390972 |
| Chloroplast (class) | 0.053195158 | 0.068638074 | 0.438426928 | 0.500468474 |
| Corynebacterium | 0.015070687 | 0.056129162 | 0.788325725 | 0.851856511 |
| Bacteroides | 0.014892149 | 0.063265499 | 0.813932199 | 0.871555717 |
| Eikenella | 0.009320694 | 0.0602182 | 0.87701308 | 0.923207775 |
| Capnocytophaga | 0.008184415 | 0.054695618 | 0.881058306 | 0.923207775 |
| Enterobacteriaceae (family) | 0.003258803 | 0.063674937 | 0.959195186 | 0.975316113 |
| Solobacterium | 0.00156382 | 0.054148234 | 0.976961209 | 0.985102553 |
| Burkholderiales (order) | 0.000916362 | 0.062919191 | 0.988383576 | 0.988383576 |
| Cryptobacterium | -0.004577788 | 0.060807294 | 0.93999693 | 0.963895157 |
| Acinetobacter | -0.008767459 | 0.064972662 | 0.892688509 | 0.923207775 |
| Moraxella | -0.009274279 | 0.064371213 | 0.885479833 | 0.923207775 |
| Cardiobacterium | -0.015084763 | 0.056226709 | 0.788495283 | 0.851856511 |
| Howardella | -0.016968645 | 0.060936528 | 0.78069896 | 0.851856511 |
| Ottowia | -0.019599636 | 0.064217521 | 0.760295988 | 0.843998298 |
| Neisseriaceae (family) | -0.026189493 | 0.063287092 | 0.679118637 | 0.760864399 |
| Peptoniphilus | -0.041848919 | 0.06385034 | 0.512416131 | 0.579461232 |
| Oxalobacteraceae (family) | -0.052418205 | 0.063346438 | 0.408333444 | 0.470555683 |
| Saccharibacteria (phylum) | -0.05417447 | 0.056059431 | 0.333915283 | 0.396115189 |
| Neisseria | -0.057488673 | 0.067343744 | 0.393336509 | 0.457631899 |
| Leptotrichia | -0.066060739 | 0.053462797 | 0.216651055 | 0.267497731 |
| Alloscardovia | -0.073870529 | 0.063876361 | 0.247638586 | 0.296675929 |
| Christensenellaceae_R-7_group | -0.081886923 | 0.063431476 | 0.197212137 | 0.248569464 |
| Scardovia | -0.083451573 | 0.066659843 | 0.210694966 | 0.26282568 |
| Veillonellaceae (family) | -0.085406236 | 0.061562348 | 0.165495515 | 0.217662579 |
| Mobiluncus | -0.086342485 | 0.063110878 | 0.17174111 | 0.223448111 |
| Lachnoanaerobaculum | -0.087782685 | 0.053687238 | 0.102100859 | 0.145343576 |
| Ruminococcaceae_UCG-002 | -0.09045606 | 0.063220013 | 0.153035478 | 0.205747698 |
| Candidate_division_SR1 (phylum) | -0.097977973 | 0.065542026 | 0.135044286 | 0.185685893 |
| Pseudomonas | -0.099837788 | 0.064312311 | 0.120900596 | 0.168149105 |
| Ralstonia | -0.100106663 | 0.063149661 | 0.113248906 | 0.159338576 |
| Stenotrophomonas | -0.108579827 | 0.064475556 | 0.092681779 | 0.135114401 |
| Abiotrophia | -0.112818692 | 0.063732014 | 0.076777884 | 0.114692888 |
| **Stomatobaculum** | **-0.119768457** | **0.054273754** | **0.027379877** | **0.047852543** |
| Bifidobacterium | -0.123800664 | 0.064625513 | 0.055507727 | 0.085018164 |
| **Ruminococcaceae_UCG-014** | **-0.125206295** | **0.057337199** | **0.029039067** | **0.048801765** |
| **Kingella** | **-0.129260244** | **0.058833875** | **0.028078765** | **0.047852543** |
| Cloacibacterium | -0.133439311 | 0.065333082 | 0.041443046 | 0.066861448 |
| **Gemella** | **-0.134207974** | **0.056577527** | **0.017724692** | **0.032495268** |
| **Prevotella_7** | **-0.134246675** | **0.05251245** | **0.010603198** | **0.020516263** |
| Ruminococcus_2 | -0.135312788 | 0.063653571 | 0.033958662 | 0.055527001 |
| **Curvibacter** | **-0.144007719** | **0.063330341** | **0.023377125** | **0.042218391** |
| **Streptococcus** | **-0.144612942** | **0.050887254** | **0.004503726** | **0.010092374** |
| **Lactobacillus** | **-0.147729326** | **0.073691051** | **0.045094873** | **0.070863372** |
| **Butyrivibrio_2** | **-0.150723934** | **0.059490036** | **0.011353683** | **0.021135317** |
| **Atopobium** | **-0.154390159** | **0.054327637** | **0.004504035** | **0.010092374** |
| **Candidatus_Saccharimonas** | **-0.161333606** | **0.060186236** | **0.007421996** | **0.015221382** |
| **Mollicutes_RF9 (order)** | **-0.161346247** | **0.063197078** | **0.010756116** | **0.020516263** |
| **Lachnospiraceae (family)** | **-0.161749569** | **0.061220344** | **0.008274372** | **0.016413098** |
| **Bergeyella** | **-0.161791864** | **0.057712907** | **0.005077181** | **0.010970338** |
| **Mannheimia** | **-0.167502883** | **0.063004554** | **0.007947487** | **0.016027432** |
| **Alysiella** | **-0.174011885** | **0.06185798** | **0.005003234** | **0.010970338** |
| **Oribacterium** | **-0.175301317** | **0.053277886** | **0.001008012** | **0.002772033** |
| **Granulicatella** | **-0.178748261** | **0.052421348** | **0.000655186** | **0.001981937** |
| **Actinomyces** | **-0.186988194** | **0.049813004** | **0.000176151** | **0.000592062** |
| **Prevotella_6** | **-0.188262137** | **0.058985788** | **0.001424028** | **0.003666116** |
| **Comamonadaceae (family)** | **-0.19739212** | **0.064880085** | **0.002405655** | **0.005940494** |
| **Haemophilus** | **-0.199002625** | **0.062163524** | **0.001376871** | **0.003666116** |
| **Veillonella** | **-0.209789919** | **0.048913901** | **1.82896E-05** | **7.13966E-05** |
| **Parascardovia** | **-0.211604574** | **0.063705514** | **0.000922753** | **0.002596583** |
| **Escherichia/Shigella** | **-0.214631773** | **0.064160529** | **0.000865256** | **0.002492762** |
| **Lautropia** | **-0.239123439** | **0.059991557** | **6.85753E-05** | **0.000244048** |
| **Megasphaera** | **-0.247366532** | **0.060655151** | **4.61258E-05** | **0.000174413** |
| **Rothia** | **-0.251560824** | **0.054432799** | **3.90604E-06** | **1.89052E-05** |
| **Gracilibacteria (phylum)** | **-0.272209758** | **0.062119533** | **1.28053E-05** | **5.38619E-05** |
| **Actinobacillus** | **-0.292435039** | **0.064522191** | **6.05217E-06** | **2.81659E-05** |
| **Staphylococcus** | **-0.318370778** | **0.063156957** | **5.55722E-07** | **2.92358E-06** |

FDR: false discovery rate. Bolded taxa indicate statistically significant differential abundance. For periodontal disease differential abundance analysis, positive log-fold change values indicated taxa that were enriched in moderate/severe periodontal disease while negative log-fold change values indicated taxa that were enriched in healthy/mild periodontal health.
